# Different factors limit early‐ and late‐season windows of opportunity for monarch development

**DOI:** 10.1002/ece3.9039

**Published:** 2022-07-11

**Authors:** Louie H. Yang, Karen Swan, Eric Bastin, Jessica Aguilar, Meredith Cenzer, Andrew Codd, Natalie Gonzalez, Tracie Hayes, August Higgins, Xang Lor, Chido Macharaga, Marshall McMunn, Kenya Oto, Nicholas Winarto, Darren Wong, Tabatha Yang, Numan Afridi, Sarah Aguilar, Amelia Allison, Arden Ambrose‐Winters, Edwin Amescua, Mattias Apse, Nancy Avoce, Kirstin Bastin, Emily Bolander, Jessica Burroughs, Cristian Cabrera, Madeline Candy, Ariana Cavett, Melina Cavett, Lemuel Chang, Miles Claret, Delaney Coleman, Jacob Concha, Paxson Danzer, Joe DaRosa, Audrey Dufresne, Claire Duisenberg, Allyson Earl, Emily Eckey, Maddie English, Alexander Espejo, Erika Faith, Amy Fang, Alejandro Gamez, Jackelin Garcini, Julie Garcini, Giancarlo Gilbert‐Igelsrud, Kelly Goedde‐Matthews, Sarah Grahn, Paloma Guerra, Vanessa Guerra, Madison Hagedorn, Katie Hall, Griffin Hall, Jake Hammond, Cody Hargadon, Victoria Henley, Sarah Hinesley, Celeste Jacobs, Camille Johnson, Tattiana Johnson, Zachary Johnson, Emma Juchau, Celeste Kaplan, Andrew Katznelson, Ronja Keeley, Tatum Kubik, Theodore Lam, Chalinee Lansing, Andrea Lara, Vivian Le, Breana Lee, Kyra Lee, Maddy Lemmo, Scott Lucio, Angela Luo, Salman Malakzay, Luke Mangney, Joseph Martin, Wade Matern, Byron McConnell, Maya McHale, Giulia McIsaac, Carolanne McLennan, Stephanie Milbrodt, Mohammed Mohammed, Morgan Mooney‐McCarthy, Laura Morgan, Clare Mullin, Sarah Needles, Kayla Nunes, Fiona O'Keeffe, Olivia O'Keeffe, Geoffrey Osgood, Jessica Padilla, Sabina Padilla, Isabella Palacio, Verio Panelli, Kendal Paulson, Jace Pearson, Tate Perez, Brenda Phrakonekham, Iason Pitsillides, Alex Preisler, Nicholas Preisler, Hailey Ramirez, Sylvan Ransom, Camille Renaud, Tracy Rocha, Haley Saris, Ryan Schemrich, Lyla Schoenig, Sophia Sears, Anand Sharma, Jessica Siu, Maddie Spangler, Shaili Standefer, Kelly Strickland, Makaila Stritzel, Emily Talbert, Sage Taylor, Emma Thomsen, Katrina Toups, Kyle Tran, Hong Tran, Maraia Tuqiri, Sara Valdes, George VanVorhis, Sandy Vue, Shauna Wallace, Johnna Whipple, Paja Yang, Meg Ye, David Yo, Yichao Zeng

**Affiliations:** ^1^ Department of Entomology and Nematology University of California Davis California USA; ^2^ Center for Land‐Based Learning Woodland California USA; ^3^ Davis Senior High School Davis California USA

**Keywords:** *Asclepias*, *Danaus plexippus*, ecological crunch, heatwaves, monarch–milkweed interactions, phenological mismatch, phenology‐ontogeny landscape, reproductive window of opportunity, seasonal fitness landscape, seasonal host plant limitation, seasonal window of opportunity, sequential hypotheses

## Abstract

Seasonal windows of opportunity are intervals within a year that provide improved prospects for growth, survival, or reproduction. However, few studies have sufficient temporal resolution to examine how multiple factors combine to constrain the seasonal timing and extent of developmental opportunities. Here, we document seasonal changes in milkweed (*Asclepias fascicularis*)–monarch (*Danaus plexippus*) interactions with high resolution throughout the last three breeding seasons prior to a precipitous single‐year decline in the western monarch population. Our results show early‐ and late‐season windows of opportunity for monarch recruitment that were constrained by different combinations of factors. Early‐season windows of opportunity were characterized by high egg densities and low survival on a select subset of host plants, consistent with the hypothesis that early‐spring migrant female monarchs select earlier‐emerging plants to balance a seasonal trade‐off between increasing host plant quantity and decreasing host plant quality. Late‐season windows of opportunity were coincident with the initiation of host plant senescence, and caterpillar success was negatively correlated with heatwave exposure, consistent with the hypothesis that late‐season windows were constrained by plant defense traits and thermal stress. Throughout this study, climatic and microclimatic variations played a foundational role in the timing and success of monarch developmental windows by affecting bottom‐up, top‐down, and abiotic limitations. More exposed microclimates were associated with higher developmental success during cooler conditions, and more shaded microclimates were associated with higher developmental success during warmer conditions, suggesting that habitat heterogeneity could buffer the effects of climatic variation. Together, these findings show an important dimension of seasonal change in milkweed–monarch interactions and illustrate how different biotic and abiotic factors can limit the developmental success of monarchs across the breeding season. These results also suggest the potential for seasonal sequences of favorable or unfavorable conditions across the breeding range to strongly affect monarch population dynamics.

## Graphical abstract

Few studies have observed species interactions with sufficient temporal resolution to examine how multiple factors combine to constrain the seasonal timing and extent of developmental opportunities. Our results show early‐ and late‐season windows of opportunity for monarch recruitment and indicate that these windows were constrained by different combinations of factors. These findings show an important dimension of seasonal change in milkweed‐monarch interactions and suggest the potential for seasonal sequences of favorable or unfavorable conditions across the breeding range to strongly affect monarch population dynamics.

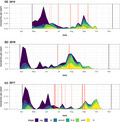

## 1 INTRODUCTION

Seasonal windows of opportunity are intervals within a year that provide improved prospects for growth, survival, or reproduction (Yang & Cenzer, 2020). These seasonal windows reflect a favorable combination of biotic and abiotic factors in space and time, including periods of increased resource availability (e.g., Ogilvie et al., 2017; Visser et al., 2006), reduced predation pressure (e.g., Rasmussen & Rudolf, 2016; Urban, 2007), or more favorable climatic conditions (e.g., Bale et al., 2002; Hunter, 1993). Although the terminology has varied, seasonal windows of opportunity have long been recognized across a wide range of systems (Bale et al., 2002; Elton, 1927; Farzan & Yang, 2018; Hunter, 1993; Ogilvie et al., 2017; Rasmussen & Rudolf, 2016; Urban, 2007; Visser et al., 2006; Yang & Rudolf, 2010). Conceptually, seasonal windows of opportunity represent a qualitative analog of the peaks in a continuous seasonal fitness landscape (Farzan & Yang, 2018; Yang & Cenzer, 2020; Yang & Rudolf, 2010), and recognize that seasonal periods of increased fitness commonly result from the combined effects of multiple bottom‐up, top‐down and abiotic factors that change over time (e.g., Farzan & Yang, 2018; Yang & Cenzer, 2020). These concepts are also fundamental to the phenological match‐mismatch hypothesis (Cushing, 1990): The match‐mismatch hypothesis represents a specific case where the window of opportunity for a focal consumer depends on the temporal availability of its resource (Kharouba & Wolkovich, 2020). More broadly, seasonal windows of opportunity represent a temporally explicit extension of the Hutchinsonian niche concept (Hutchinson, 1957; Yang, 2020), analogous to a phenological niche (Post, 2019; Wolkovich & Cleland, 2011, 2014).

Seasonal windows of opportunity are constrained by bottom‐up, top‐down, and abiotic factors. Efforts to quantify the relative contribution of these factors address a fundamental paradigm in ecology and suggest testable predictions about the factors that structure populations and communities. However, in addition to their independent contributions, these factors could also have more complex, interactive, and temporally specific effects on seasonal windows of opportunity. For example, their importance could vary across or within years, or multiple limiting factors could combine sequentially in time. Studying these processes requires a temporally explicit approach: the examination of shorter intervals of time to better understand the dynamics of changing systems (Yang, 2020). Temporally explicit approaches involve a quantitative change in the frequency of observations but have the potential to facilitate qualitative improvements in our understanding of seasonally variable systems.

Here, we present a temporally explicit, high‐resolution study of milkweed (*Asclepias fascicularis*)–monarch (*Danaus plexippus*) interactions observed across 3 years. The goal of this study is to better understand the factors that limit seasonal windows of opportunity for monarch caterpillars. The population of monarch butterflies in western North America largely overwinters in aggregations along the California coast (Lane, 1993; Leong et al., 2004; Tuskes & Brower, 1978; Yang et al., 2016); in the late winter, these populations become reproductively active and migrate inland from their coastal overwintering sites to find suitable host plants (Dingle et al., 2005; Nagano et al., 1993). This migratory breeding season population expands across their western North American range over multiple generations before largely returning to coastal overwintering populations in the late summer and early fall (Dingle et al., 2005; Yang et al., 2016). Previous experimental studies have suggested that early‐season and late‐season windows of opportunity on narrow‐leaved milkweed (*Asclepias fascicularis*) in the California Central Valley could result from seasonal patterns of growth and defensive trait expression, which affect both the quantity and quality of host plants available to migrating monarchs (Yang et al., 2020; Yang & Cenzer, 2020). These studies suggest that phenological mismatches could create seasonal host plant limitations, especially if periods of high oviposition densities coincide with small host plant sizes (Yang & Cenzer, 2020). However, previous experimental studies were unable to assess three key factors that could affect monarch developmental success in nature: (1) the effects of inter‐ and intra‐annual climatic variation, (2) the effects of seasonal variation in monarch densities, and (3) the effects of microhabitat heterogeneity. Although it is clear that monarch developmental success can be strongly limited by bottom‐up (Flockhart et al., 2015; Nail, Stenoien, et al., 2015; Pleasants & Oberhauser, 2013; Yang et al., 2020; Zalucki & Lammers, 2010), top‐down (Altizer & Oberhauser, 1999; De Anda & Oberhauser, 2015; Hermann et al., 2019; Oberhauser, 2012; Oberhauser et al., 2015; Prysby, 2004) and abiotic (Nail, Batalden, et al., 2015; Stevens & Frey, 2010; York & Oberhauser, 2002; Zalucki, 1982) factors, few studies have examined how multiple factors combine to limit wild milkweed–monarch interactions across the breeding season in a high‐resolution, temporally explicit framework.

This study aimed to address three specific questions: (1) How do the developmental prospects of monarchs vary in time, within‐ and across years? (2) How do the combined effects of bottom‐up, top‐down, and abiotic factors interact with seasonal variation in monarch density to constrain the timing and extent of seasonal windows of opportunity? and (3) How do climatic variation and microhabitat heterogeneity affect these constraints?

## 2 METHODS

### 2.1 Field site establishment

**FIGURE A1:**
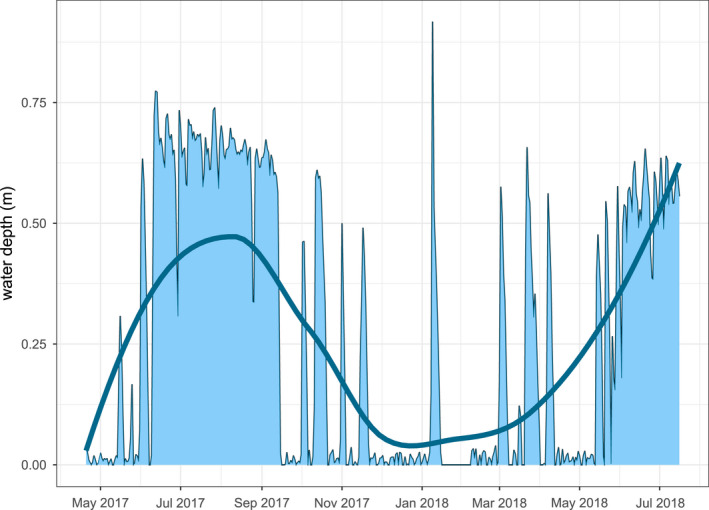
Seasonal water depth in the North Davis irrigation channel. This channel conveys agricultural irrigation and storm runoff water and shows a pattern of increased flows in the summer months when irrigation activity is greatest and intermittent flood events from natural precipitation events in the winter. The thick blue line represents a locally estimated scatterplot smoothing (LOESS) moving regression fit

In December 2013, we planted a population of 318 individually identified narrow‐leaved milkweed (*Asclepias fascicularis*) at approximately 6.1 m intervals in an approximately 2 km linear transect adjacent to a seasonal irrigation channel (38°34′18.5″N 121°45′29.6″W) in Davis, CA (Yolo County) USA. These milkweeds were propagated from seedlings using locally collected seeds (Hedgerow Farms, Winters, CA USA). These milkweeds were established as part of a larger effort to create a California riparian plant community including grasses, rushes, sedges (e.g., *Bromus carinatus*, *Carex* spp., *Distichlis spicata*, *Elymus* spp., *Hordeum brachyantherum*, *Juncus* spp., *Leymus triticoides, Muhlenbergia rigens, Nassella pulchra, and Poa secunda)*, shrubs (e.g., *Ceanothus cuneatus, Cephalanthus occidentalis, Heteromeles arbutifolia, Rhamnus californica, and Symphoricarpos albus)*, and trees (e.g., *Eucalyptus* spp., *Fraxinus latifolia, Platanus racemosa, Populus fremontii, Quercus* spp., and *Salix* spp.). This riparian corridor runs adjacent to agricultural fields and a suburban neighborhood, carrying runoff water with a seasonal pattern of generally increased flow during summer irrigation periods and immediately following winter precipitation events (Figure A1). As a result, this site combines several elements representative of the California Central Valley at a landscape scale.

### 2.2 Environmental data

We obtained daily temperature maxima, daily temperature minima, and daily precipitation total data for Davis, CA (Global Historical Climatology Network Station USC00042294) over the 20‐year period from 1998 to 2018 from the NOAA Climate Data Online Portal (National Centers for Environmental Information, 2018). To create a complete dataset, we imputed missing daily values (1.2% of the available dataset) using a bootstrapping algorithm implemented in the Amelia II package in R (Honaker et al., 2011; R Core Team, 2020) using priors based on daily means and standard deviations.

In addition to this dataset of daily temperature minima and maxima, we also analyzed a second dataset of sub‐hourly temperature observations (approximately every 20 min) from the same source to inform a thermal accumulation model of developmental degrees‐days and thermal stress exposure for monarchs in the early and late growing season each year. We define the early season as days 90–180 (approximately the end of March to the end of June) and the late season as days 180–270 (approximately the end of June to the end of September) each year. Developmental degree‐days for monarchs were calculated using a lower developmental baseline temperature of 11.5°C (Zalucki, 1982) with linear positive thermal accumulation up to 36°C (Masters et al., 1988; York & Oberhauser, 2002). While early studies conducted under constant temperature conditions showed upper developmental thresholds of 28–29°C for monarch development (Barker & Herman, 1976; Zalucki, 1982), subsequent studies have shown that cooler nighttime temperatures allow for continued development under daytime temperatures up to 36°C (York & Oberhauser, 2002), with sublethal thermal stress emerging at temperatures exceeding 38°C (Nail, Batalden, et al., 2015). Thus, we defined developmental degree‐days as the product of exposure duration and degrees above 11.5°C up to 36°C, and thermal stress degree‐minutes as the product of exposure duration and degrees exceeding the 38°C threshold. Finally, we calculated exposures to temperatures exceeding 42°C, a threshold that has been shown to cause mortality in a very high proportion of monarch caterpillars after a 12 h exposure (Nail, Batalden, et al., 2015). We present both the duration of exposures above this lethal threshold and the accumulation of lethal degree‐minutes, defined as the product of exposure duration and degrees greater than 42°C.

We also developed a model of thermal accumulation in narrow‐leaved milkweed, using a developmental baseline of 11.5°C (based on unpublished data). For the milkweed model, we calculated the accumulation of thermal exposure each year between day 1 and day 163, the day when 75% of milkweed plants exceeded total 50 cm stem length study‐wide across all 3 years.

We also obtained state‐level drought data for the period from 1998 to 2018 from the National Integrated Drought Information System at drought.gov (NIDIS, 2019), which classifies the percent of the state under five levels of drought severity over time.

At the site level, we assessed the canopy openness above each milkweed using digital image analysis in ImageJ (Abramoff et al., 2004) of hemispheric photographs taken at approximately 1 m height in July 2016. We also measured representative seasonal changes in the water depth of the irrigation channel at 30‐min intervals between April 20, 2017, and July 16, 2018, using a data‐logging water depth meter (Onset HOBO U20L). These data were corrected for daily changes in atmospheric pressure using a dataset from the nearest available location (Sacramento Airport, CA, USA) obtained from the NOAA Climate Data Online Portal (National Centers for Environmental Information, 2018).

### 2.3 Monitoring milkweed–monarch interactions

We collected data at approximately weekly intervals (*mean observation interval*: 8.2 days/observation in 2015, 7.1 days/observation in 2016, and 7.0 days/observation in 2017) throughout each growing season (*observation period*: April 27 to November 15 in 2015, March 31 to November 4 in 2016, and April 5 to November 9 in 2017) in 2015, 2016 and 2017. Across the 3‐year study, an average of 94% of the study population was measured each week, and this metric of data completeness increased each year (86% per week in 2015, 97% per week in 2016, and 98% per week in 2017). For each observation on each milkweed (7919 observations in 2015, 9973 observations in 2016, 10,006 observations in 2017, 27,898 observations in total), we used a standardized protocol that included assessments of plant status (presence of emergent stems, percentage of nonsenescent tissue, percentage of leaf area removed by herbivores), measurements of plant size (number of stems >5 cm, mean stem length, mean stem diameter), counts of milkweed reproduction (number of open floral umbels, number of nonsenescent seed pods longer than 1 cm), and measurements of any monarch eggs or caterpillars present (number of monarchs eggs, number of monarchs of each larval instar, larval length). Percentages of nonsenescent tissue and leaf area removed were estimated visually, measurements of stem length were taken with meter sticks to the nearest cm, and measurements of stem diameter and larval length were measured using dial calipers to the nearest 0.1 mm. Finally, participants collected additional notes, including observations of the surrounding predator and herbivore community.

Measurements were collected by 159 observers (36 observers in 2015, 53 observers in 2016, and 89 observers in 2017) during a total of 2027 person‐hours in the field (679 person‐hours in 2015, 659 person‐hours in 2016, and 689 person‐hours in 2017). Most observations were collected by participants in the Monitoring Milkweed–Monarch Interactions for Learning and Conservation (MMMILC) Project. Louie Yang provided hands‐on, in‐person training in milkweed‐monarch biology, data collection, and data entry protocols in partnership with the Environmental Science internship program led by Eric Bastin at Davis Senior High School (Davis, CA USA) and the Growing Green internship program led by Karen Swan at the Center for Land‐based Learning (Woodland, CA USA). Training sessions represented 4.5 to 6.5 h of in‐person training, sometimes spread over 2–3 days or provided during a single day‐long workshop event. Training included detailed guidance in identifying and measuring milkweed, identifying and measuring monarch eggs and larvae, and data collection and data entry protocols. Participants were evaluated based on their knowledge of monarch and milkweed biology (e.g., species and stage identification, life history, general ecology) and project‐specific skills and protocols (e.g., reading dial calipers, recording data in the field, entering data online, visually estimating percent herbivory). Participants were required to successfully complete an evaluation of knowledge and skills before collecting data for the project. For each week of data collection, available participants were randomly assigned to teams of two to three observers. Each team was randomly assigned to a set of approximately 30–60 consecutively numbered milkweed plants, with sets evenly distributed across the transect. Each team carried a standard field kit including an illustrated milkweed field guide (Rea et al., 2003) and customized, site‐specific laminated photo identification guides for narrow‐leaved milkweed, monarch instars, and other locally common milkweed‐associated arthropods. Team members alternated between taking measurements and recording data. In teams of three, the third team member documented observations and photographs in a publicly accessible blog. This protocol was designed to facilitate interspersion and minimize the potential for the confounded observer and team effects within and across weeks.

Data were collected with datasheets in the field and entered into shared Google spreadsheets within 24 h of each data collection effort. Undergraduate and graduate student mentors with previous experience in milkweed‐monarch research provided guidance in the field during the first weeks of each field season to facilitate data quality and continuity as new participants transitioned into the project. Eric Bastin and Karen Swan provided additional weekly guidance throughout each season, and Louie Yang was available throughout the summer and was present during many weekly data entry sessions to answer additional questions that arose. Participants entered data that they recorded in the field to facilitate handwriting interpretation. We downloaded and analyzed data periodically throughout each field season, using a preliminary R script (R Core Team, 2014) to identify emerging data quality issues and provide rapid data summaries to participants. In 2016 and 2017, we also used the data validation tools in the Google spreadsheet and weekly comparisons of the physical datasheets and the online dataset to prevent data entry errors.

We excluded measurements, which were likely to have resulted from data entry errors from the analysis. These included 0.11% of stem diameter measurements (96 of 86,363) that exceeded 15 mm (Z‐score >5.65) and 0.02% of stem length measurements (21 of 86,945) that exceeded 150 cm (Z‐score >4.78). In most cases, these data appear to have resulted from missing decimal points. Excluding these data likely had a negligible effect on the overall analysis because they represent a very small proportion of the overall dataset and because our analysis used multiple measurements per plant as subsamples to calculate an observation‐level mean for each milkweed at each visit. We did not detect data entry errors in other metrics of plant or monarch development.

### 2.4 Analysis of milkweed growth and phenology

Because narrow‐leaved milkweed growth with multiple lateral stems, we used two metrics to estimate plant size. *Total stem length* estimated the cumulative length of stems on the branching growth form of narrow‐leaved milkweed, while *total cross‐sectional stem area* provides a cumulative metric of stem thickness. The total stem length of each plant at each observation was estimated as the product of the mean observed individual stem length and the total stem count. The total cross‐sectional stem area of each plant at each observation was similarly estimated as the product of the mean observed cross‐sectional stem area and the total stem count. The total stem count included all shoots (main stems and lateral stems) with a nonsenescent length greater than 5 cm. We calculated the mean stem length and mean stem diameter from measurements of 10 haphazardly selected stems per plant unless fewer stems than 10 stems were available. These stems were chosen to provide a representative subsample of the stem length distribution on each plant.

We aggregated the resulting dataset on annual and weekly scales to summarize all available milkweed and monarch metrics each week and for each of the 3 years in the study. All analyses were conducted in R (R Core Team, 2020) using the *tidyverse* package (Wickham et al., 2019).

The emergence phenology of milkweed was quantified as the mean date when plants exceeded a total stem length of 5 cm. To identify the period of increased host plant biomass (i.e., the viable season length) each year, we defined an interval bounded by a threshold of plant size (the date when the population mean exceeded a threshold total stem length of 50 cm) and a threshold of plant senescence (the date when the population mean fell below 80 percent greenness). These boundary conditions place approximate and qualitative milestones informed by previous studies in this system (Yang et al., 2020) to quantify a period of increased host plant viability for monarch development.

We analyzed the role of canopy openness as a microhabitat variable affecting milkweed emergence, growth, and phenology using linear and generalized linear models (GLMs) with canopy openness, year, and their interaction as predictors. Our model of milkweed emergence phenology used the day of year when each plant exceeded a total stem length of 5 cm as the response variable. In this and all subsequent linear models, assumptions of residual normality and homoscedasticity were assessed using Q‐Q plots and residual plots. A second linear model examined the day of year when milkweeds exceed a total stem length greater than 50 cm. A third growth model used the maximum total stem length attained by each plant as a response variable. This model used a gamma conditional distribution with a log‐link function. The gamma distribution is flexibly and appropriately applied to positive, continuous data with an approximately log‐normal distribution. A fourth, final linear model examined the day of year when plants first showed greenness values less than 80%. In all models, nonsignificant (α = 0.05) interaction terms were removed before examining the main effects. When significant interaction effects were present, we examined simple effects separately.

### 2.5 Analysis of monarch growth and phenology

Weekly monarch observation counts provide information about the relative abundance of monarchs across each year and allow comparisons between years. We examined annual and seasonal differences in egg and caterpillar observation counts considering the effects of year, season (early vs. late), and their interaction using separate GLMs with Poisson conditional distributions and log‐link functions. We chose the Poisson distribution a priori due to the count‐based (positive integer) response variables.

In order to visualize seasonal patterns in the survivorship of monarchs, we examined the ratio between the maximum number of fifth instar caterpillars observed per week divided by the maximum number of eggs observed per week (fifth instar: egg) for each season × year combination. This ratio provides a relative metric indicative of the proportion of observed eggs that are later observed as fifth instar larvae. We found qualitatively similar patterns when considering ratios of other stages (fifth instar: first instar and fifth instar: second instar).

To assess the potential for seasonal variation in oviposition site selection based on milkweed size, we compared the mean size of milkweeds with and without monarch eggs present each week. We quantified this comparison using a log ratio ($\log({\bar{s}}_{e}/{\bar{s}}_{0})$), where ${\bar{s}}_{e}$ represents the mean size of milkweeds with eggs present and ${\bar{s}}_{0}$ represents the mean size of milkweeds without eggs in a given week. This ratio provides a metric of apparent host plant size selectivity where positive values reflect a preference for comparatively larger host plants, while negative values reflect a preference for comparatively smaller host plants. We tested for significant deviations of this ratio from zero in each week using a Fisher–Pitman permutation test, implemented in the R package *coin* (Hothorn et al., 2008). In addition, we evaluated if the observed distributions of monarch egg and caterpillars counts per plant deviated from the random null assumption of a Poisson distribution; this test assesses the degree to which monarch observations were clumped, random or overdispersed among host plants.

We further examined the effect of canopy openness on the total annual count of monarch egg and larval observations per plant using separate Poisson GLMs with log‐link functions. These models considered canopy openness, year, and their interaction as predictors. A subsequent Poisson GLM considered canopy openness, year, season (early vs. late), and their second‐order interactions as predictors. We also examined the effects of milkweed size (maximum total stem length) and milkweed phenology (first day of each year with a total stem length greater than 50 cm) on the total annual count of monarch larval observations per plant using a Poisson GLM; both models also assessed year effects and their interactions using Type II sums of squares. We also developed a generalized additive model (GAM) that included milkweed phenology (the timing of the median size threshold) as a predictor variable for the total annual count of monarch caterpillars to assess the potential for nonlinear effects on total larval observation counts across each year. We compared this GAM model with its GLM counterpart using AIC. The AIC favored the GLM, and we report only those results.

We analyzed the notes field of our dataset to quantify the proportion of notes each week that included observations of taxa that were potential predators or competitors of monarch eggs or caterpillars during our study. We used the same approach to quantify the proportion of notes that included observations of adult monarchs each week. Observed predatory taxa were small milkweed bugs (*Lygaeus kalmii*), ladybird beetles (Coccinellidae), wasps (Vespidae), jumping spiders (Salticidae), crab spiders (Thomisidae), ants (Formicidae), hoverfly larvae (Syrphidae), lacewings (Chrysopidae), mantids (Mantodea), and earwigs (Dermaptera). Observed herbivorous taxa were oleander aphids (*Aphis nerii*), small milkweed bugs (*Lygaeus kalmii*), large milkweed bugs (*Oncopeltus fasciatus*), blue milkweed beetles (*Chrysochus cobaltinus*), milkweed longhorn beetles (*Tetraopes basalis*), planthoppers (Fulgoromorpha) and leafhoppers (Cicadellidae). Small milkweed bugs (*Lygaeus kalmii*) were counted as both predatory and herbivorous taxa due to their strongly omnivorous habits (Root, 1986). We examined two binomial GLM models to examine the effects of canopy openness, year, and their interaction on the proportion of notes that included predator or competitor observations, respectively.

In a final set of models, we evaluated the relative and combined effects of key factors hypothesized to affect egg and caterpillar observation counts. First, we evaluated a GLM considering milkweed size (maximum total stem length), thermal stress exposure (degree‐minutes ≥38°C), exposure to predators (proportion of notes with predators observed), season (early vs. late), and all pairwise interaction effects with the season. This model used a Poisson conditional distribution with a log‐link function to account for the count‐based response variable. If this analysis suggested a significant seasonal interaction effect, we subsequently compared separate models focused on the early and late seasons.

## 3 RESULTS

### 3.1 Environmental data

**FIGURE A2:**
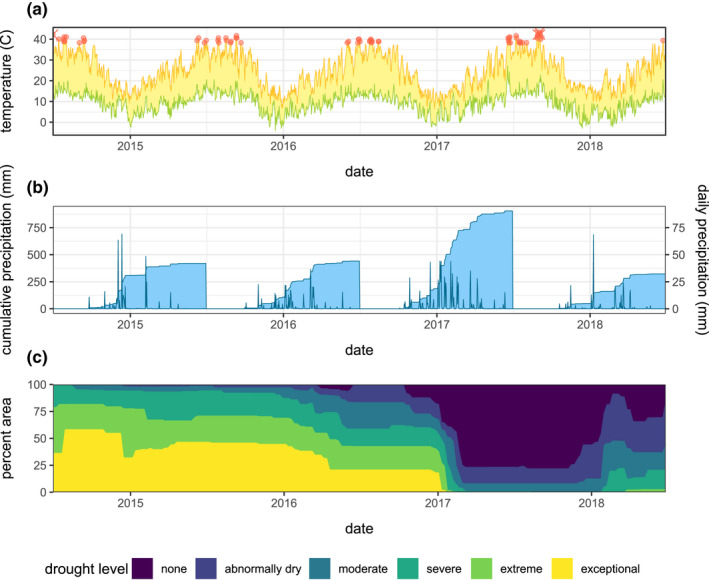
(a) Temperature and (b) precipitation in Davis, CA, USA during the study period. (a) The daily temperature minima and maxima are shown in green and orange, respectively. Days with temperature maxima ≥38°C are indicated with red points. Days with temperatures exceeding 42°C are indicated by a red ×. (b) Daily precipitation and cumulative annual precipitation. Cumulative annual precipitation is calculated between July 1 and June 30 of the succeeding year. (c) Percent of land area in CA, USA under each US Drought Monitor classification of drought severity

**FIGURE A3:**
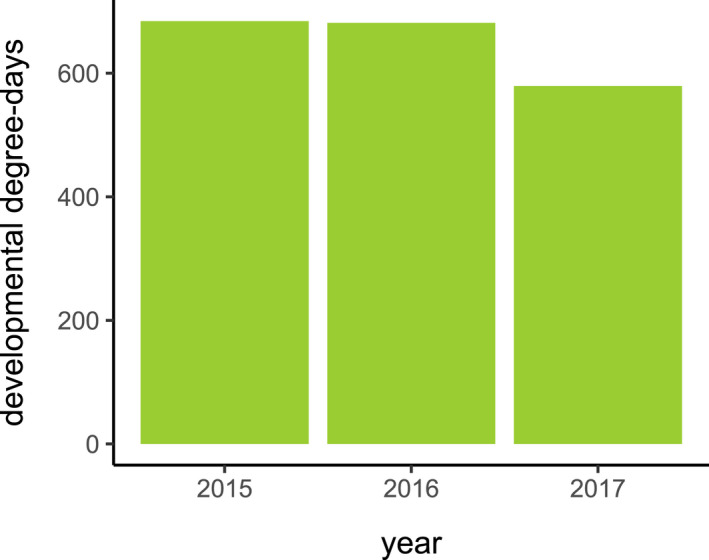
The accumulation of thermal degree days for narrow‐leaved milkweed, based on a developmental baseline of 11.5°C (unpublished data), and estimated during the period from day 1 to day 163, when 75% of plants exceeded a total stem length of 50 cm. The cooler, wetter winter preceding the 2017 growing season resulted in 15% less thermal accumulation than in each of the two preceding years

Climatic observations show a Mediterranean pattern of cool, wet winters and hot, dry summers during the study period (Figure A2). Water levels in the lower channel were consistent in the summer and intermittent in the winter (Figure A1). Daily weather observations show low annual precipitation totals for the rainy seasons leading into 2015 and 2016, with an increased frequency of precipitation events in the rainy season leading into 2017 (Figure A2b). Cumulative precipitation in the 2016–2017 wet season was 2.2 times that of the mean cumulative precipitation in 2014–2015 and 2015–2016 (Figure A2b). These local observations were consistent with a broader regional‐scale pattern that included the last 2 years (2015–2016) of a significant multi‐year drought event in California, followed by a year with rapidly ameliorating drought conditions after the relatively wet winter of 2016–2017 (Figure A2b,c). The winter preceding the 2017 season was comparatively cool and wet; our thermal accumulation model for milkweeds estimated 15% less exposure to developmentally favorable temperatures in the first 163 days of 2017 relative to 2015 or 2016 (Figure A3).

**FIGURE 1:**
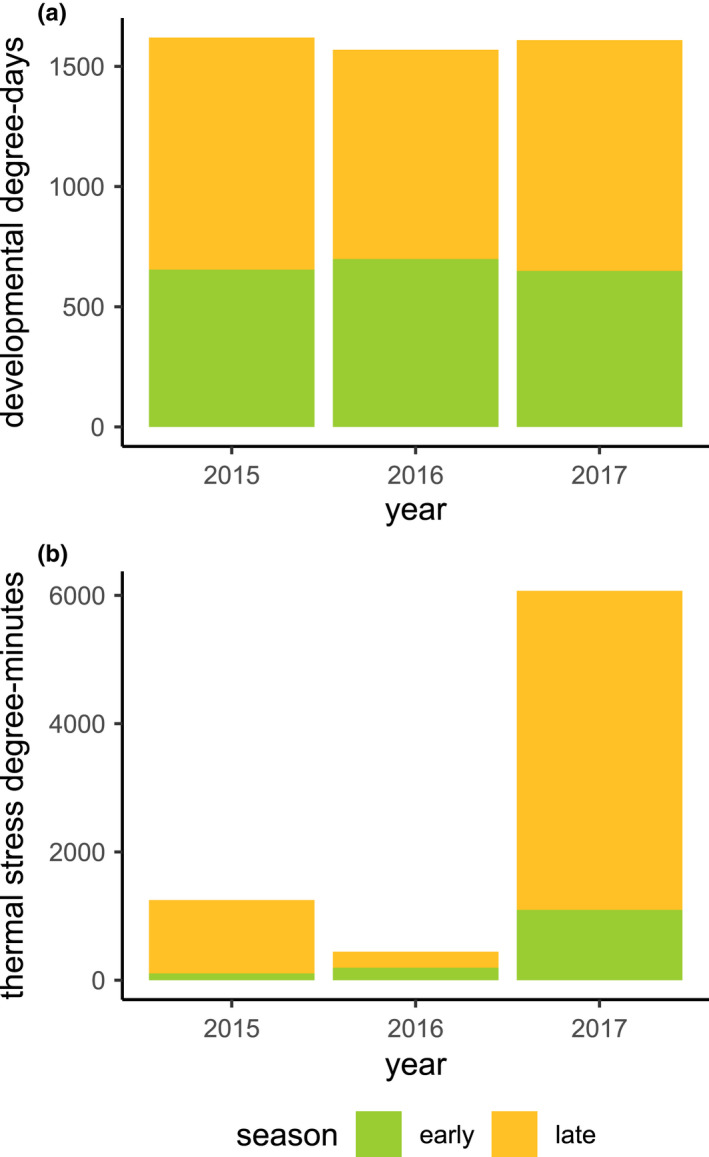
(a) Developmental degree‐days and (b) thermal stress degree‐minutes for the early (days 90–180) and late (days 180–270) growing seasons in 2015–2017. Although each year showed similar thermal accumulation across the developmentally relevant temperatures, exposure to stressful high temperatures was higher in 2017 than in 2016 or 2015

**FIGURE A4:**
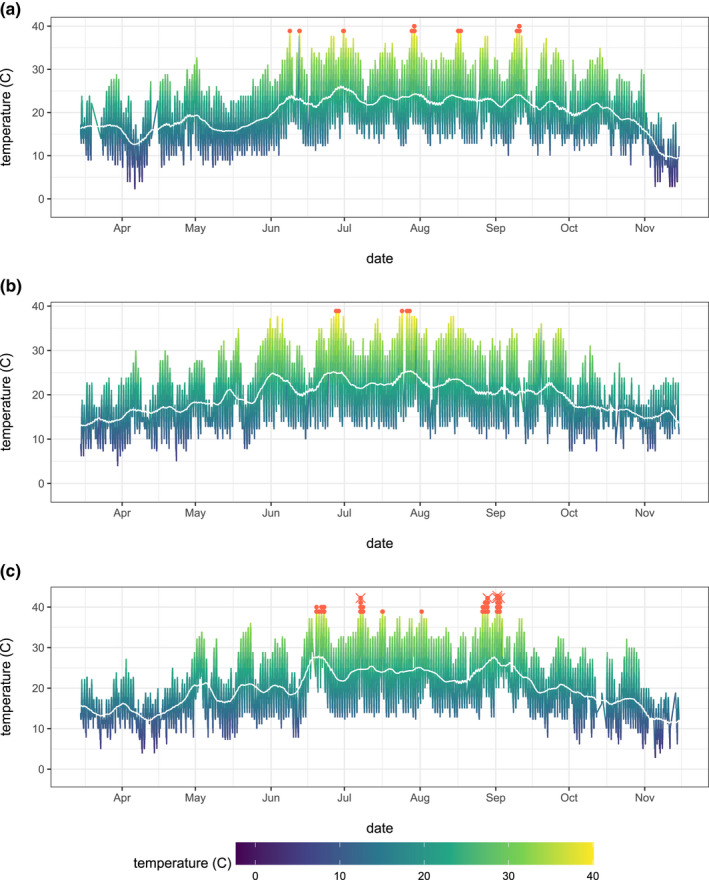
Subhourly temperature measurements in (a) 2015, (b) 2016, and (c) 2017. The colored line indicates the temperature at approximately 20‐min intervals. The white line indicates the 10‐day rolling mean, and observations >38°C are marked with a red point. Observations with temperatures exceeding 42°C are indicated by a red ×

However, the early and late monarch developmental periods were generally warmer in 2017 than in the two previous years (Figures 1 and A2). All 3 years of this study showed similar thermal accumulation in the developmentally relevant range for monarchs between 11.5–36°C (York & Oberhauser, 2002; Zalucki, 1982), with 40% more developmental degree‐days in late seasons compared with early seasons (Figure 1a). However, the accumulation of stressful degree‐days (≥38°C) was substantially greater in the late season compared with the early season (971% greater in 2015, 127% greater in 2016, 353% greater in 2017, 355% greater overall). Exposure to stressful high temperatures also varied strongly among years, with much greater exposure to stressful temperatures in 2017 (Figure 1b). When comparing daily high temperatures, 2017 experienced 19 days above 38°C, compared with 15 such days in 2015 and 14 in 2016 (Figure 1b). These differences were larger when assessed on a sub‐hourly scale, where the accumulation of stressful degree‐minutes was 386% higher in 2017 than in 2015 and 1266% higher in 2017 than in 2016. By comparison, the late season of 2016 showed a notable lack of thermal stress accumulation, experiencing only 249 stressful degree‐minutes, compared with 1142 stressful degree‐minutes in the late season of 2015 and 4972 stressful degree‐minutes in the late season of 2017 (Figures 1b and A4). In addition, 3 days in the late season of 2017 recorded temperatures exceeding 42°C (Figure A4); this was the only year with temperatures exceeding the lethal threshold. The total duration of exposure to temperatures exceeding 42°C was 500 min in 2017, accumulating 131 lethal degree‐minutes.

### 3.2 Milkweed survival, growth, and phenology

**FIGURE 2:**
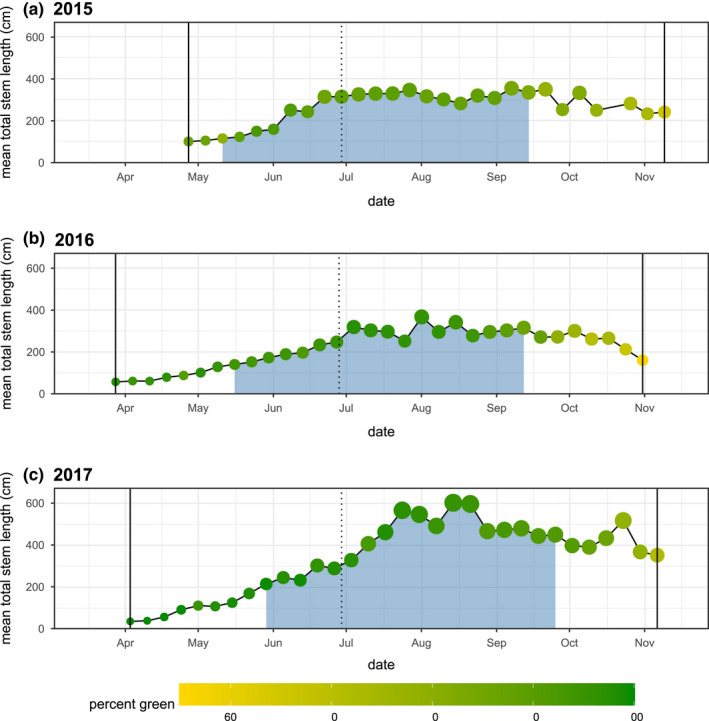
Mean milkweed total stem length and percent green at weekly intervals across three growing seasons. Point size and the vertical axis indicate the weekly mean plant size, and point color indicates the weekly mean percent green. The blue region represents a period of increased host plant availability for monarch development bounded by the mean date when plants exceeded 75 cm total stem length on the left and the mean date when percent green was declined below 80% on the right. Solid vertical lines indicate the start and end of observations at each season. The dotted vertical line represents day 180, which is used to separate the early and late season in these analyses.

**FIGURE A5:**
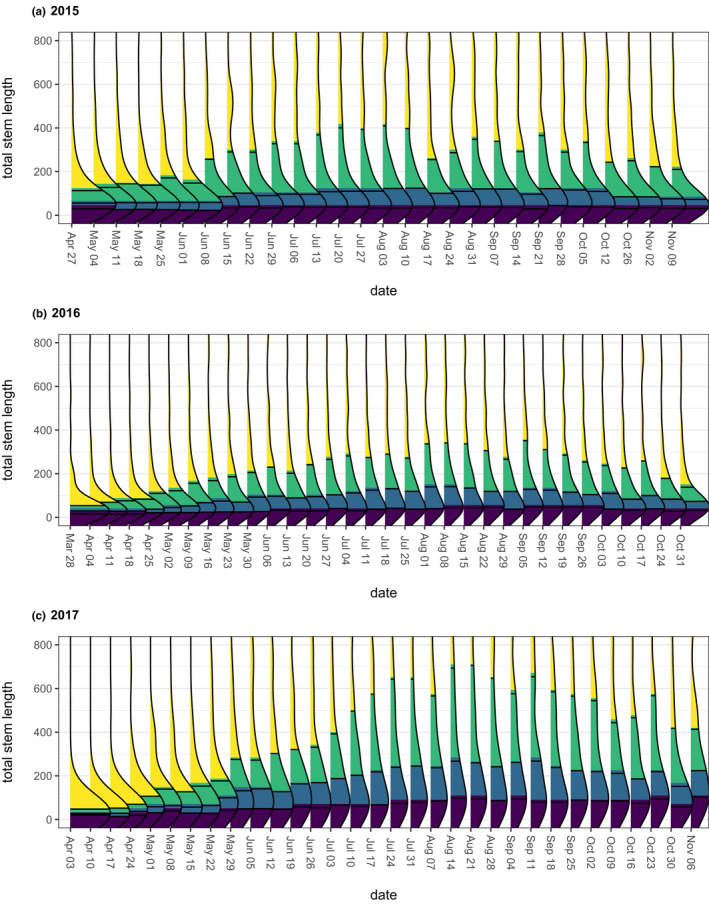
Distribution of total stem lengths at weekly time slices through (a) 2015, (b) 2016, and (c) 2017. Colors represent quartiles. Across all years, seasonal changes in the median plant size correspond with larger increases in the upper quartiles in the late season. In the post‐drought 2017 seasons, increases in the median plant size were associated with broader plant size distributions. Note that these three panels share a common vertical axis but are scaled to the observation period of each year on the horizontal axis

The number of surviving emerged plants declined over the 3‐year study, from 281 (88.3%) in 2015, to 238 (75%) in 2016 to 226 (71%) in 2017. However, an increasing proportion of the surviving plants attained a total stem length exceeding 50 cm across these same years: 137 (49% of 281) in 2015, 144 (61% of 238) in 2016, and 175 (77% of 226). The growth of milkweeds changed dramatically in 2017 following the rainy winter of 2016–2017. Milkweeds in 2017 attained sizes (maximum weekly mean total stem lengths) that were 70% larger than in 2015, and 64% larger than in 2016 (Figure 2), and the variance of the plant size distribution also increased (Figure A5).

**FIGURE 3:**
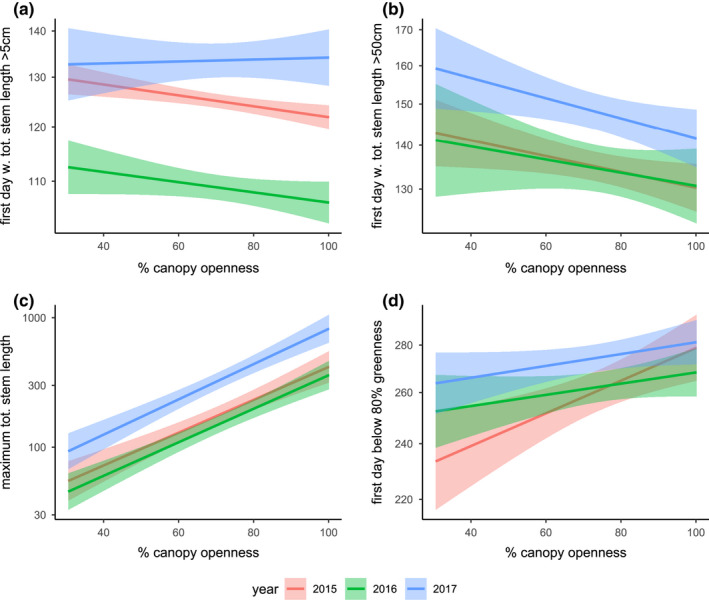
Effects of canopy openness on (a) the phenology of milkweed emergence, (b) the timing of milkweed growth, (c) maximum total stem length and (d) timing of senescence. Canopy openness was generally associated with earlier milkweed emergence, earlier growth to a viable host plant size, and larger maximum milkweed sizes across the season

On May 5, 2017, City of Davis maintenance staff unintentionally mowed this site, damaging several plants in this population. However, most plants in the population were below the height of the mower blades at this point in the growing season; only 6% of the viable plant population showed reduced total stem lengths immediately after the mowing event, and we did not observe a substantial decline in population mean total stem length immediately afterwards (Figure 3). Thus, this disturbance probably had a relatively small effect on the phenology of the overall milkweed population in our study, though it may have delayed the assembly of the predator community (Haan & Landis, 2019).

**FIGURE A6:**
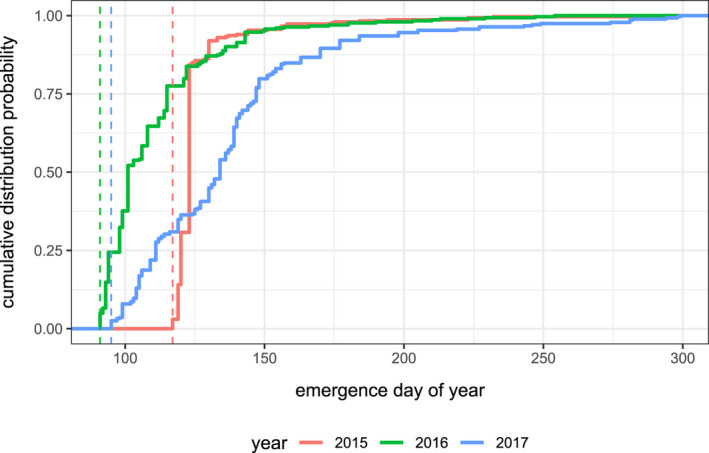
Cumulative distribution of milkweed emergence dates in 2015, 2016, and 2017. Accounting for the later start of the observation period in 2015, the emergence phenology was likely similar in 2015 and 2016, while 2017 shows a flatter, delayed phenology

The timing of milkweed emergence varied strongly among years (*p* < .0001). Milkweed emerged earliest in 2016 (mean emergence day 110) and nearly four weeks later in 2017 (mean emergence day 137). The mean emergence day of year for 2015 was intermediate (day 125), but these emergence observations were limited by the relatively late start of the observation period in 2015 (day 117, compared with day 91 in 2016 and day 95 in 2017) and likely underestimate the phenological advancement of the 2015 growing season. Cumulative distribution plots of milkweed emergence (Figure A6) suggest that 2015 likely showed emergence phenology similar to 2016, as both years showed quantitatively similar size distributions in the week following the initiation of observations in 2015, with a subsequent cumulative distribution pattern that is qualitatively distinct from the flattering pattern observed in 2017.

Across all years, milkweeds emerged marginally earlier in microhabitats with greater canopy openness (*p* = .08753, Figure 3a); this effect of canopy openness did not differ significantly among years (canopy openness × year, *p* = .2514), although 2017 showed a qualitatively different positive effect coefficient (Figure 3a).

**FIGURE A7:**
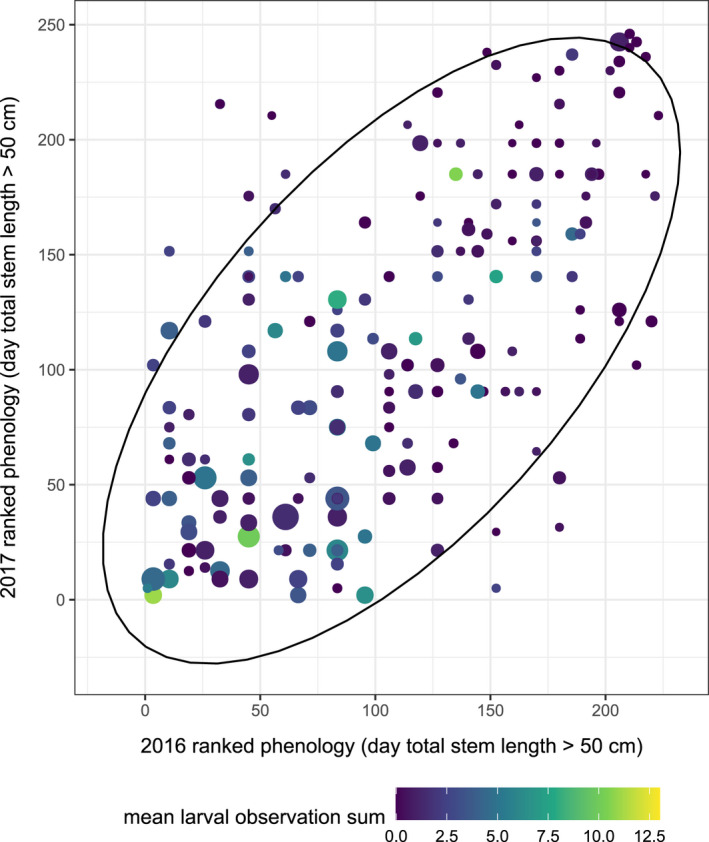
Correlation in 2016 and 2017 ranked milkweed phenologies measured as the first day each year when a plant’s total stem length exceeded 50 cm. The significant correlation (*r* = .59, *p* < .0001) suggests that the relative phenology of milkweed plants was consistent between these two years; early plants tended to be early in both years. Early plants also tended to be larger (indicated with point size) and generally supported more larval monarch observations (indicated with point color) than later growing, smaller plants

Milkweeds in locations with greater canopy openness grew to a viable host plant size threshold (50 cm total stem length) earlier (*p* = .0021, Figure 3b) and attained larger maximum sizes across the year (*p* < .0001, Figure 3c). In this model, each percent of canopy openness advanced the timing of this size threshold by 0.23 days. The timing of this threshold varied by year (*p* < .0001); milkweeds attained a total stem length of 50 cm earliest in 2015, followed by 2016 (3.4 days later) and 2017 (15.2 days later), but the effect of canopy openness on growth phenology did not vary significantly among years (canopy openness × year, *p* = .73). The model of the maximum total size used a log‐link function, so the exponent of the model coefficients yields a multiplicative effect size: Each percent of increased canopy openness predicted a 3.6% increase in maximum stem length. Milkweeds were largest in 2017 and smallest in 2016 (year, *p* < .0001), but the effect of canopy size on maximum milkweed size did not vary by year (canopy openness × year, *p* = .23) and the ranked phenology of milkweed plants was highly correlated between years (*r* = .59, *p* < .0001, Figure A7).

**FIGURE A8:**
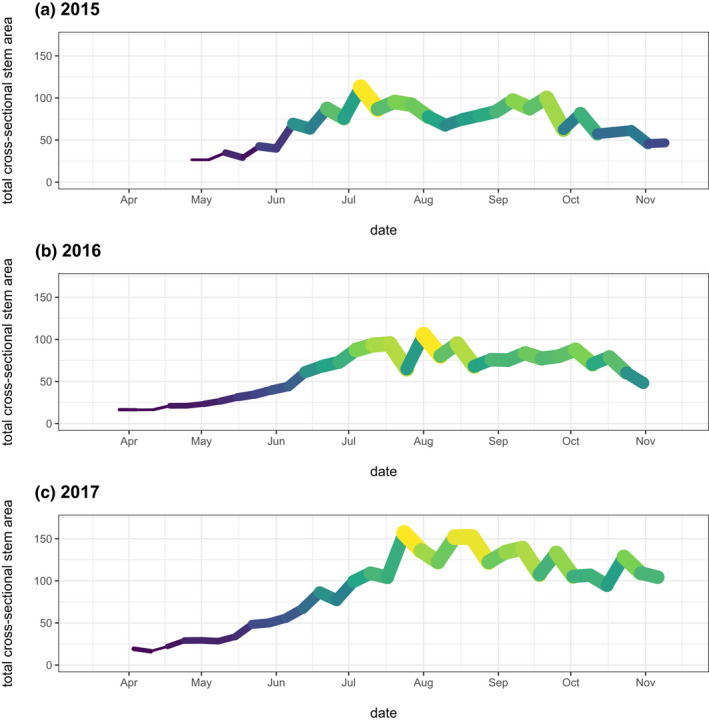
Weekly mean total cross‐sectional stem areas across (a) 2015, (b) 2016, and (c) 2017. Color is scaled within each year to emphasize the timing and duration of the highest stem areas within each year. Color is mapped to total cross‐sectional stem area and rescaled within each year to emphasize the seasonal peak of each year

**FIGURE A9:**
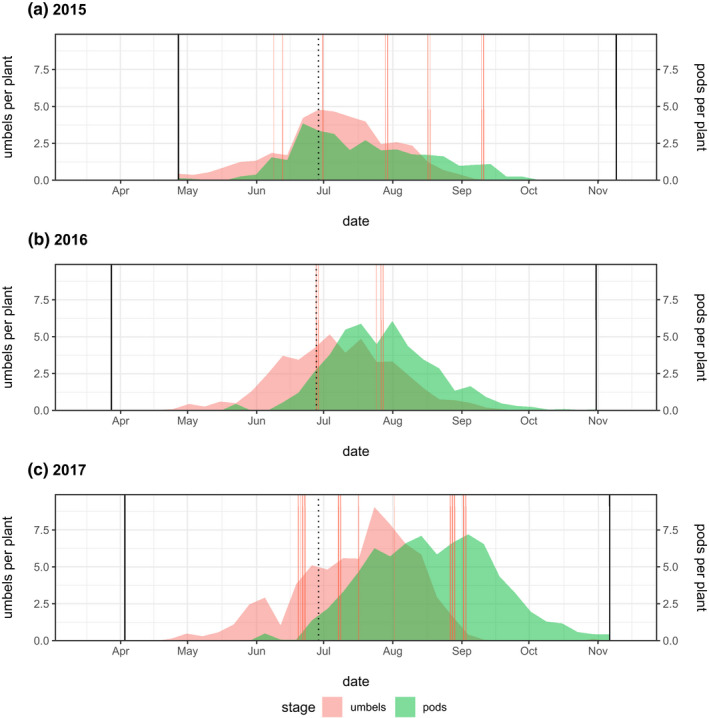
Phenology of milkweed flowering (weekly mean umbel count per plant) and seed production (weekly mean seed pod count per plant) in (a) 2015, (b) 2016, and (c) 2017. Milkweed reproductive phenology was delayed in 2017 compared with 2015 and 2016. Solid red vertical lines indicate periods when the temperature exceeded 38°C

We observed similar patterns of milkweed phenology with measures of total stem cross‐sectional area and reproduction (flowering and seed pod production). Stem cross‐sectional area was dynamic across each season (Figure A8), but the annual mean was markedly higher overall in 2017 (90.0 mm^2^) than in either 2015 or 2016 (68 and 60 mm^2^, respectively). The reproductive phenology of milkweeds was advanced in the two drought years (2015 and 2016) and delayed in the postdrought year (2017, Figure A9). The peak floral display was approximately 3 weeks later in 2017 (the week of July 24) than in 2015 or 2016 (the weeks of June 29 and July 4, respectively). The production of seed pods showed an even more pronounced pattern of delayed phenology in each successive year: peak pod counts occurred during the week of June 22, 2015, August 1, 2016, and September 4, 2017 (Figure A9).

On average, milkweeds in 2017 senesced 11 days later than in 2016, and 10 days later than in 2015 (*p* = .006, Figure 2). Milkweeds in more open canopy environments generally senesced later (*p* = .0003, Figure 3d), and this effect did not differ significantly between years (canopy openness × year, *p* = .14).

### 3.3 Monarch phenology, growth, and herbivory

#### 3.3.1 Between and within‐year patterns of monarch observations

**FIGURE 4:**
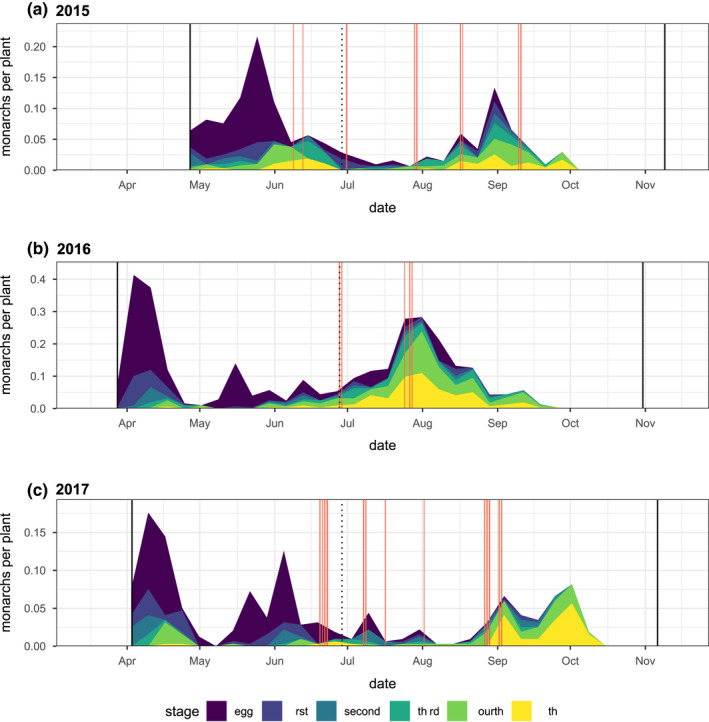
Monarch observation density per milkweed host plant across three growing seasons. Colors indicate egg or larval instar. Solid black vertical lines indicate the start and end of observations each season. The dotted vertical line represents day 180, which is used to separate the early and late season. Solid red vertical lines indicate periods when the temperature exceeded 38°C

**FIGURE 5:**
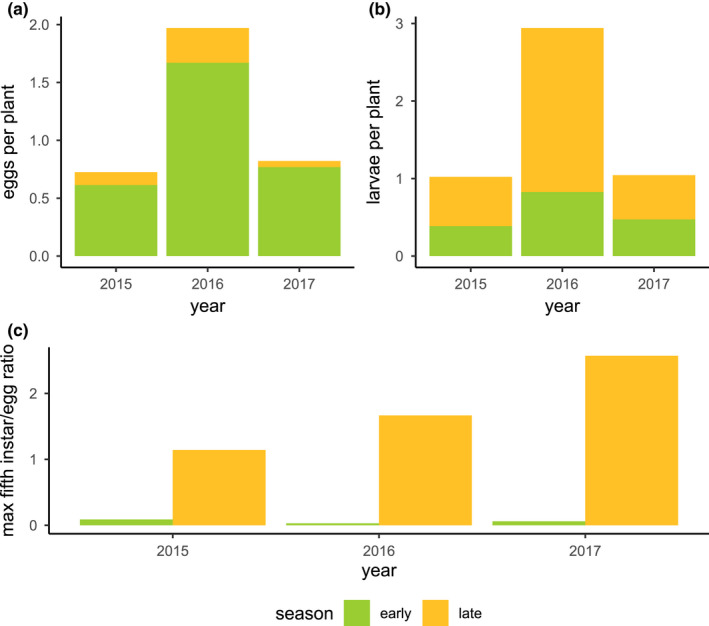
(a) Monarch egg and (b) caterpillar observations per emerged plant in the early (before day 180) and late (after and including day 180) season each year. (c) The ratios of maximum weekly observed counts of fifth instar caterpillars relative to eggs in the early and late growing season likely reflect relative rates of survival to pupation in the early and late season each year

We documented 674 weekly observations of monarch eggs and 997 weekly observations of monarch caterpillars across the 3 years of this study. Monarchs were most numerous in 2016 (Figures 4 and 5). We observed 2.7 times as many monarch eggs in 2016 as in 2015 and 2.2 times as many as in 2017. We observed 3.0 times as many caterpillars in 2016 as in 2015, and 2.5 times as many as in 2017. Separated by year and normalized by the total number of emerged plants each year, we observed 137 eggs and 193 caterpillars (0.49 egg and 0.69 caterpillar observations per plant) in 2015, 369 eggs and 576 caterpillars (1.55 egg and 2.42 caterpillar observations per plant) in 2016 and 168 eggs and 226 caterpillars (0.74 egg and 1.0 caterpillar observations per plant) in 2017.

**FIGURE A10:**
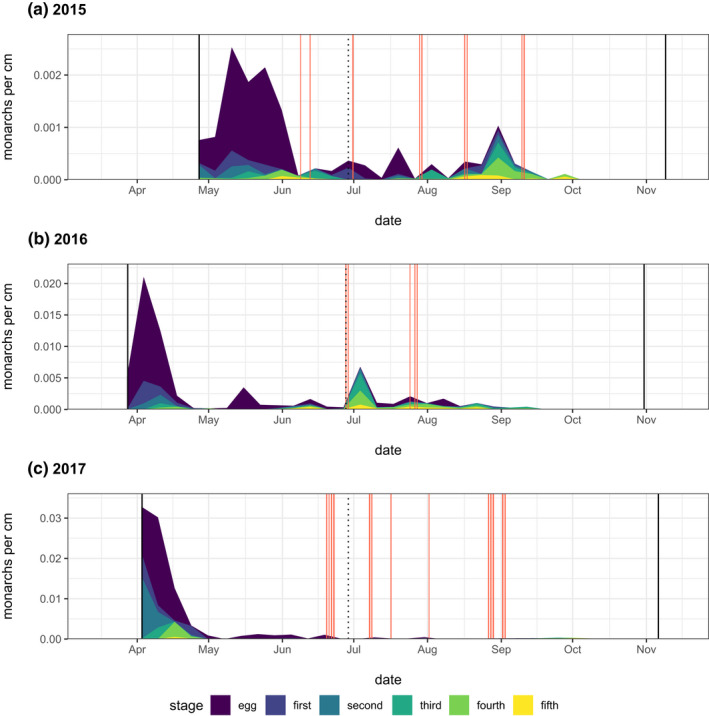
Monarch observation density per cm of milkweed stem length across three growing seasons. Normalizing by plant size allows visualization of potential early season milkweed limitation. Solid black vertical lines indicate the start and end of observations each season. The dotted vertical line represents day 180, which is used to separate the early and late season in these analyses. Solid red vertical lines indicate periods when the temperature exceeded 38°C

**FIGURE A11:**
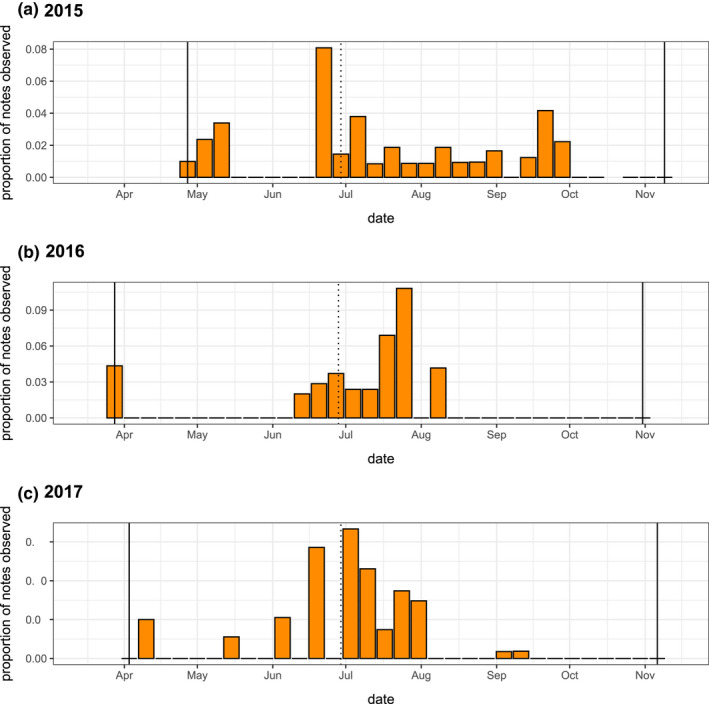
Seasonal patterns of adult monarch observations in (a) 2015, (b) 2016, and (c) 2017

The seasonal pattern of monarch observations (Figure 4) showed early and late seasonal windows of opportunity on narrow‐leaved milkweed host plants. Early‐season peaks of egg deposition and late‐season peaks of larval observation were evident in all years (Figure 4), though 2015 and 2017 showed substantially lower peak densities compared with 2016. Normalizing observation counts by a seasonally varying metric of plant size (total stem length) emphasizes periods of high monarch density relative to host plant availability (Figure A10). These early and late periods of increased monarch egg and caterpillar observations contrasted with the mid‐summer seasonal pattern of adult monarch observations at our site (Figure A11).

**FIGURE A12:**
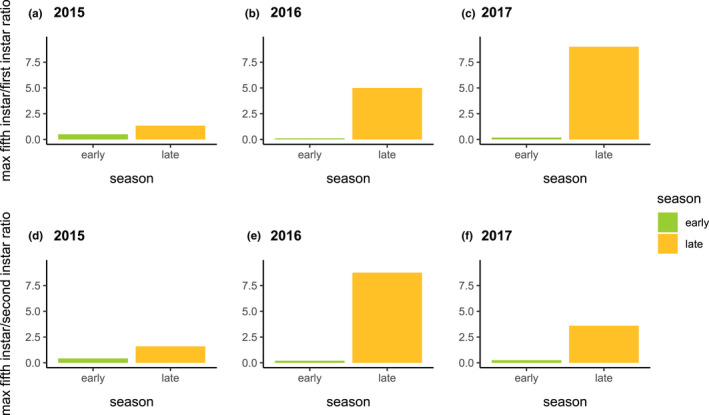
Early versus late season differences in the observation ratios of fifth instar caterpillars relative to first and second instar caterpillars are consistent with those seen with eggs (Figure 5)

In every year of this study, the majority of egg observations were in the early season (Figure 5a), and the majority of caterpillar observations were in the late season (Figure 5b). A GLM of egg counts per plant that included year (2015, 2016, and 2017) and season (early and late) as explanatory factors showed a significant interaction (*p* = .01192), though year‐specific models showed significantly higher egg observations in the early season every year (2015: *p* < .00001, 2016: *p* < .00001, and 2017: *p* < .0001). In comparison, a GLM of caterpillar counts showed a significant interaction between year and season (*p* < .0001), with year‐specific models showing significantly more larvae in the late (vs. early) season in 2015 (*p* = .0006) and 2016 (*p* < .0001) but not in 2017 (*p* = .16). Combining all 3 years, 86.2% of egg observations were in the early season, while 67.6% of caterpillar observations were in the late season. The ratio of fifth instar larvae to egg observations differed markedly between the early and late seasons in all 3 years of this study (Figures 5c and A12). In 2015, 2016, and 2017, this ratio was 12.9, 55.1, and 42.4 times higher in the late season than in the early season, respectively.

#### 3.3.2 Host plant selection

**FIGURE A13:**
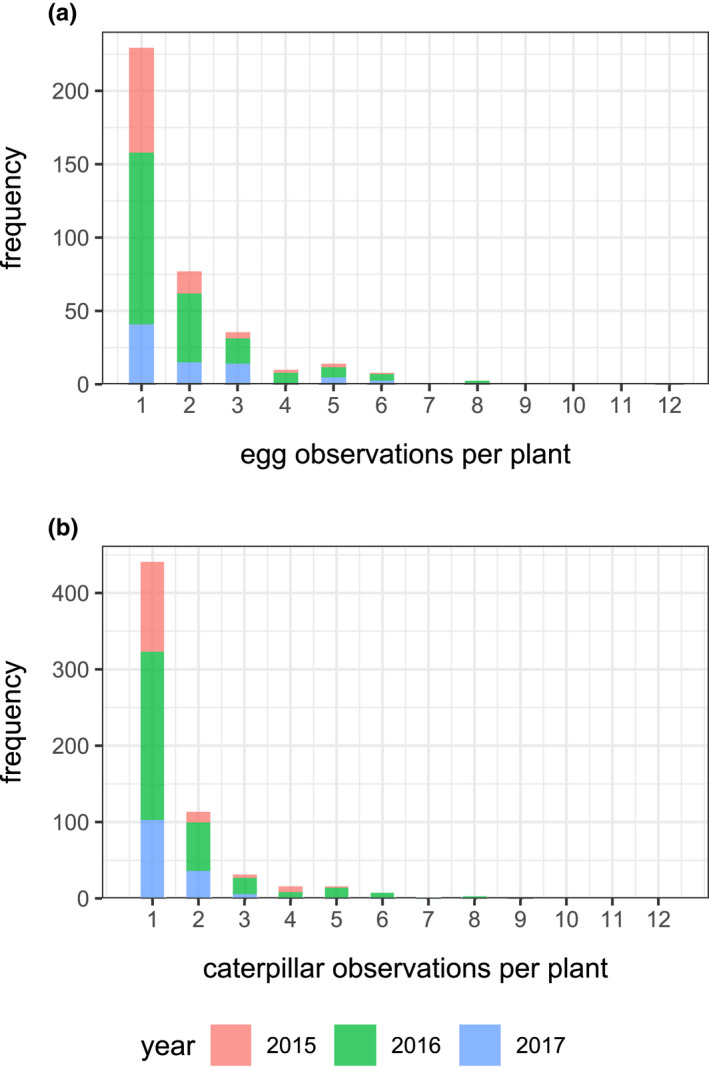
Histograms of (a) egg and (b) caterpillar densities per plant, excluding observation where eggs and caterpillars were absent

Oviposition was concentrated on a subset of selected plants. Across the entire study, 1.3% of observations included monarch eggs and 2.3% included monarch caterpillars. On average, 80% of monarch egg observations were on 16.6% of milkweeds each year (15.4% in 2015, 22.1% in 2016, and 12.2% in 2017). For observations with monarch eggs present, 61% of milkweeds had single eggs, 20% had two eggs, and the remaining 19% had three to 12 eggs present (Figure A13a). Looking at these same data from an egg perspective, 34% of eggs were observed singly, 23% were observed in pairs, and 43% were observed in densities greater than two eggs per plant. Similar patterns were observed with caterpillars (Figure A13b): Where caterpillars were present, 70% of host plants included one caterpillar, 18% included two, and the remaining 12% included three to nine caterpillars per plant. From the caterpillar perspective, 44% of caterpillars were observed singly, 23% were observed in pairs, and 33% were observed at densities between three and nine caterpillars per host plant. Egg and caterpillar counts were significantly overdistributed relative to Poisson expectations (egg, *p* < .0001; caterpillar, *p* < .0001), consistent with selective distribution of eggs and caterpillars on preferred host plants. However, when consideringly only those plants with nonzero egg or caterpillar counts, respectively, the distribution of monarch counts did not differ significantly from the random Poisson null (egg, *p* = .72; caterpillar, *p* = .26). Thus, these results suggest that ovipositing females chose host plants selectively, but they showed neither conspecific attraction nor avoidance within the set of selected plants.

**FIGURE 6:**
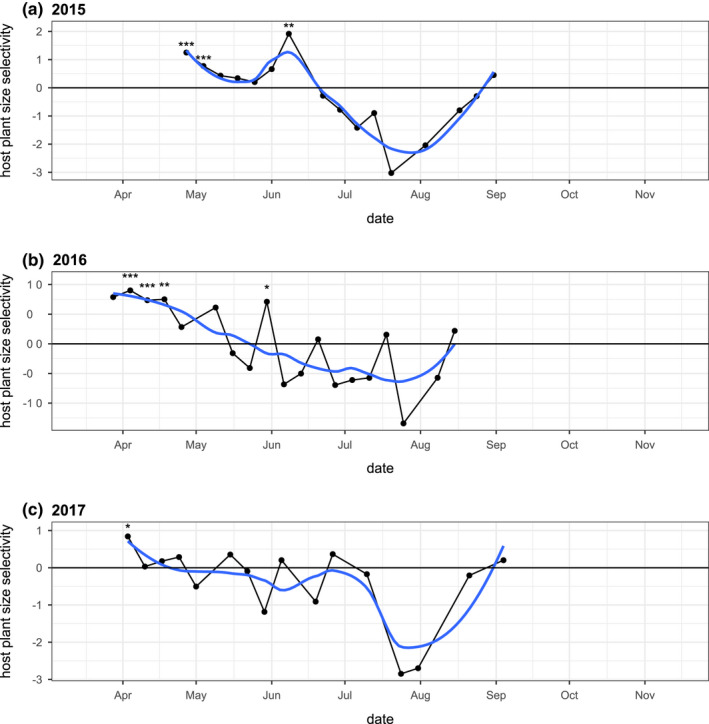
Points indicate the log ratio of the mean total stem length of milkweeds with monarch egg observations versus milkweeds without monarch egg observations for all weeks with monarch egg observations; positive values indicate apparent preference for comparatively larger plants, while negative values indicate an apparent preference for comparatively smaller plants. The blue line represents a locally estimated (LOESS) scatterplot smoothing fit. Stars indicate significant deviations from zero using a Fisher–Pitman permutation test (***p = 0 to .001; **.001–.01; *.01–.05)

In the early season, milkweeds with monarch egg observations were generally larger than milkweeds without monarch egg observations, and weekly permutational tests showed significant positive host plant size selection in the early season each year (Figure 6). As the season progressed, this host plant size selectivity eroded and eventually reversed; by the beginning of the late season, the milkweed plants with monarch egg observations tended to be smaller than milkweeds without monarch egg observations. However, the Fisher‐Pitman permutation test was unable to detect significant deviations from zero in the late season likely due to the smaller numbers of host plants with eggs present. By the end of the egg‐laying period, selectivity seemed to erode again, resulting in metrics of host plant size selectivity near zero.

#### 3.3.3 Spatiotemporal patterns and canopy openness

**FIGURE A14:**
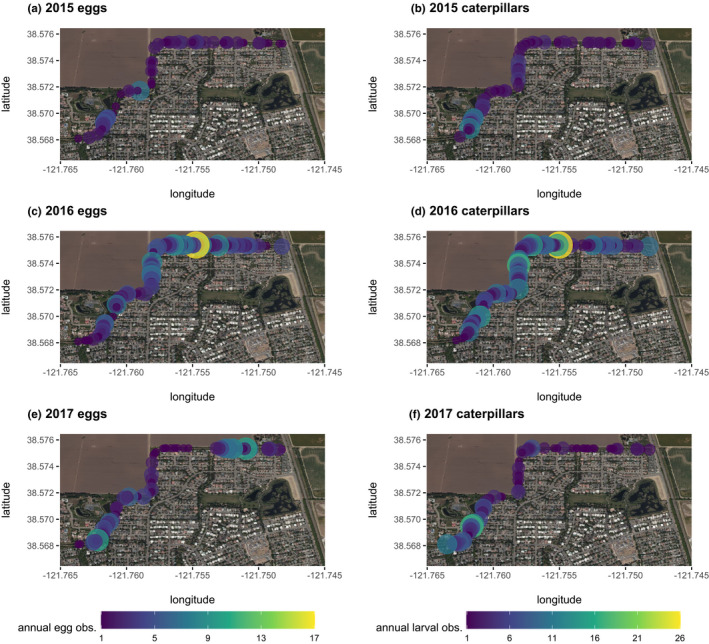
The cumulative spatial distribution of monarch egg and caterpillar observations across the study site. Points indicate the location of milkweed plants with monarch egg and caterpillar observations; size and color correspond with the annual total number of monarch observations per plant

**FIGURE A15:**
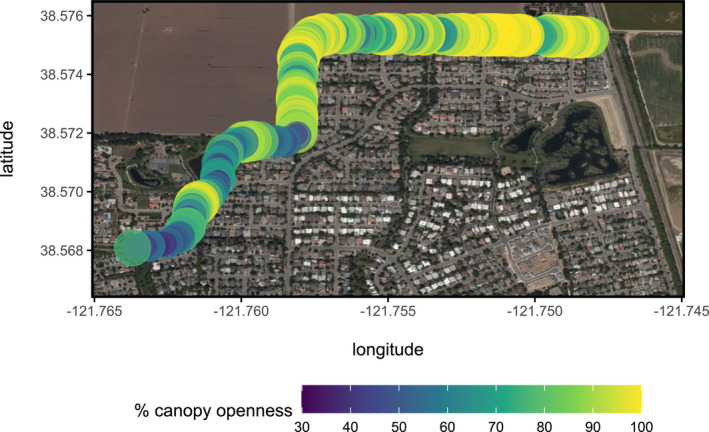
The spatial distribution of canopy openness across the study site. Points indicate the location of milkweed plants; size and color correspond with measurements of percent canopy openness above each plant

The spatial distribution of monarchs varied by year, showing year‐to‐year differences in the locations of highest oviposition activity and larval observation density (Figure A14). Both oviposition and subsequent larval observations were widely distributed across the study site in every year of the study, but the areas of greatest observation density varied from year to year. In 2015 and 2017, the density of egg and caterpillar observations was especially high in the southwestern third of the study transect while the concentration of observations was more centrally located in 2016. A map of canopy openness shows generally greater canopy cover in the southwestern third of the study site, with canopy openness increasing in the central and eastern sections of the study site (Figure A15).

**FIGURE 7:**
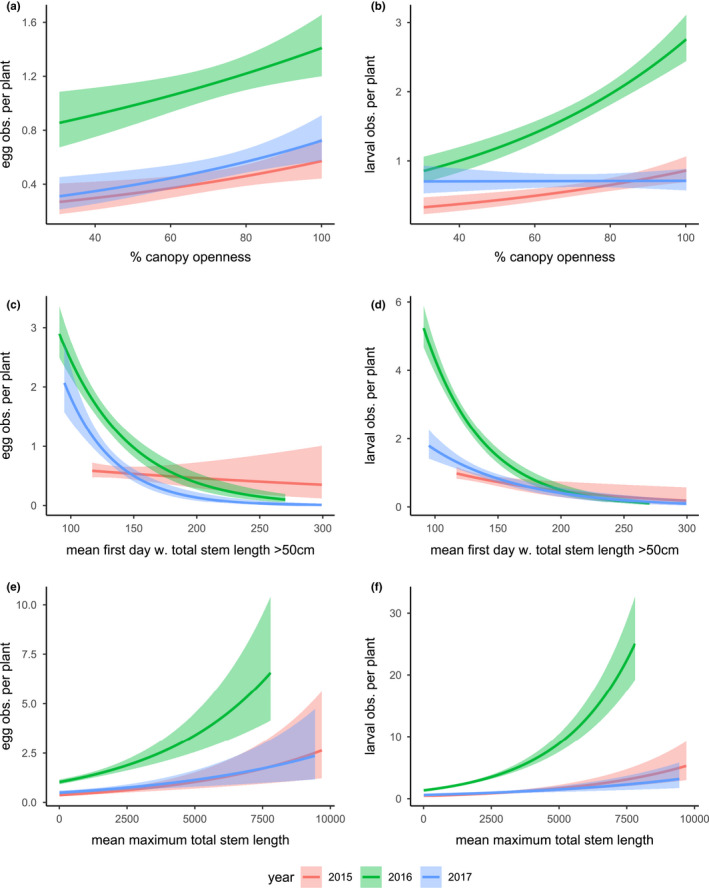
The effects of (a, b) canopy openness, (c, d) the timing of milkweed growth, and (e, f) maximum total stem length on the density of monarch egg (a, c, e) and caterpillar (b, d, e) observations per plant. Plants with more open canopies, earlier growth phenologies, and larger maximum sizes were generally associated with more monarch observations (but see *Discussion*). Shaded area around each fitted line indicates the 95% confidence interval

Milkweeds growing in microhabitats with greater canopy openness showed higher egg densities (*p* < .0001, Figure 7a). Mean egg observation densities differed by year (*p* < .0001), but the effect of canopy openness did not (*p* = .4972). Overall, each percentage increase in the canopy openness increased the expected density of egg observations by a factor of 1.009191. Because this is a multiplicative factor, this model predicts an 89% increase in egg observation densities across the range of canopy openness values observed in this study (30–100%).

Open canopy environments were also associated with higher densities of monarch caterpillar observations per plant overall (*p* = .0001, Figure 7b), and this effect was much weaker in 2017 than in the other years (canopy openness × year, *p* < .0001). In 2015, the predicted yearly caterpillar count increased by a multiplicative factor of 1.013574 (*p* = .0001) for each percentage increase in canopy openness. In 2016, this multiplicative factor increased to 1.016974 (*p* < .0001), reflecting an approximately 25% stronger effect of canopy openness in a year with greater larval monarch production overall (Figure 7d). In contrast, the effect of canopy openness was not significant (*p* = .95) in 2017, with a multiplicative effect size reduced to 1.000191. Thus, the proportional effect of canopy openness on larval observation density was more than 80‐fold greater in 2015 and 2016 than in 2017. In terms of model predictions, across the range of values observed in this study, canopy openness predicts a 222% increase in caterpillar observations in 2015, a 159% increase in caterpillar observations in 2016 but only a 1.3% increase in 2017.

In general, monarch eggs and caterpillars showed similar spatial distributions across the study. However, one notable exception was observed in 2017 where an early‐season concentration of egg observations in the more exposed eastern section of the study site was not mirrored in subsequent caterpillar observations (Figure A14e,f, Movies S1 and S2).

These relationships with canopy openness were consistent with the modeled effects of milkweed growth phenology, where plants that attained a 50 cm threshold of total stem length earlier generally supported more monarch eggs (Figure 7c) and caterpillars (Figure 7d) across the season. For egg observations, there was a significant interaction between canopy openness and year (*p* < .0001), with no significant effects detected in 2015 (*p* = .38) but strong and significant effects detected in 2016 (*p* < .0001) and 2017 (*p* < .0001). Across the interquartile range of threshold dates (day 120–163), predicted egg densities decreased by 12.6% in 2015 but declined by 120% in 2016 and 200% in 2017. For caterpillar observations, these effects varied among years and were especially strong in 2016 (canopy openness × year, *p* = .0012). Considering each year separately, our models predicted a 2.12% reduction in the larval observations for each additional day required to attain the threshold host plant size in 2016 (*p* < .0001), compared with a 0.88% reduction per day in 2015 (*p* = .0067) and a 1.4% reduction per day in 2017 (*p* < .0001). Thus, across the observed interquartile range of 50 cm threshold dates (day 120–163), our model predicts 45% higher caterpillar observation densities for early plants in 2015, 151% higher caterpillar observation densities for early plants in 2016, and 82% higher caterpillar observation densities for early plants in 2017.

**FIGURE A16:**
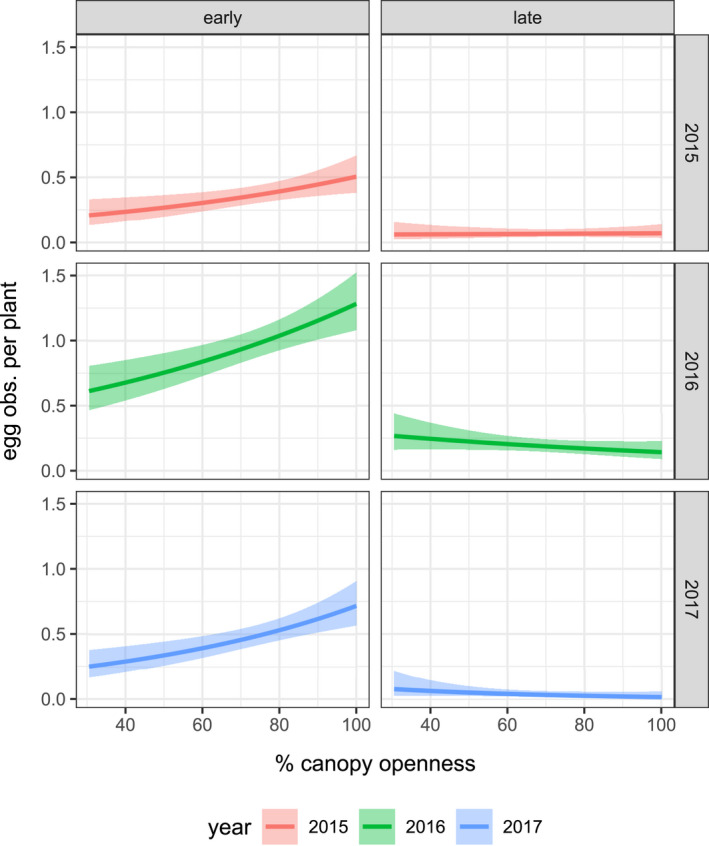
The effects of canopy openness on monarch egg densities in the early and season of each year. These analyses indicate significant positive effects in each early season, but in no significant effects in each of the late seasons. Shaded area around each fitted line indicates the 95% confidence interval

**FIGURE A17:**
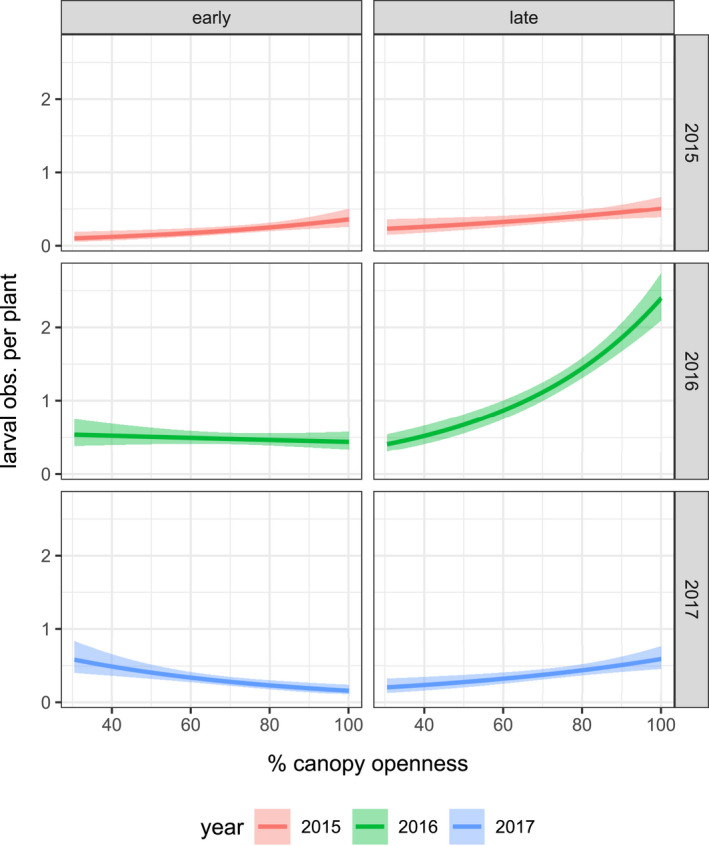
The effects of canopy openness on monarch caterpillar densities in the early and season of each year. These analyses show relatively weak and inconsistent effects in the early season of each year, followed by positive effects in the late season of each year. Shaded area around each fitted line indicates the 95% confidence interval

A model that considered the season‐specific effects of canopy openness on monarch egg observation densities indicated that the effect of canopy openness differed in the early and late seasons (canopy openness × season, *p* = .0001), with significant positive effects in each early season but no significant effects in each of the late seasons (Figure A16). By comparison, a model considering these effects on caterpillar observation densities also showed a significant canopy openness × season interaction effect (*p* < .0001), with relatively weak and inconsistent effects in the early season of each year, followed by positive effects in the late season of each year, with especially strong effects in 2016 (Figure A17).

Plants that grew to larger maximum sizes were generally associated with more monarch eggs (*p* < .0001, Figure 7e). While egg observation densities varied by year (*p* < .0001), the effect of maximum total stem length did not (max. Total stem length × year, *p* = .36). Larger milkweed plants also generally supported more monarch caterpillar observations than smaller plants (Figure 7f), but—as with the effect of milkweed phenology—this effect was especially strong in 2016 (max. Total stem length × year, *p* < .0001). In 2016, each additional cm of stem length was associated with 0.037% more monarchs over the season (*p* < .0001), compared with 0.024% in 2015 (*p* < .0001) and 0.016% in 2017 (*p* < .0001). Because these effects are multiplicative, they result in large impacts: Across the interquartile range of maximum plant sizes (62–646 cm), our model predicted the expected number of annual caterpillar observations to increase by 15% in 2015, 24% in 2016 and 10% in 2017.

#### 3.3.4 Leaf damage

**FIGURE 8:**
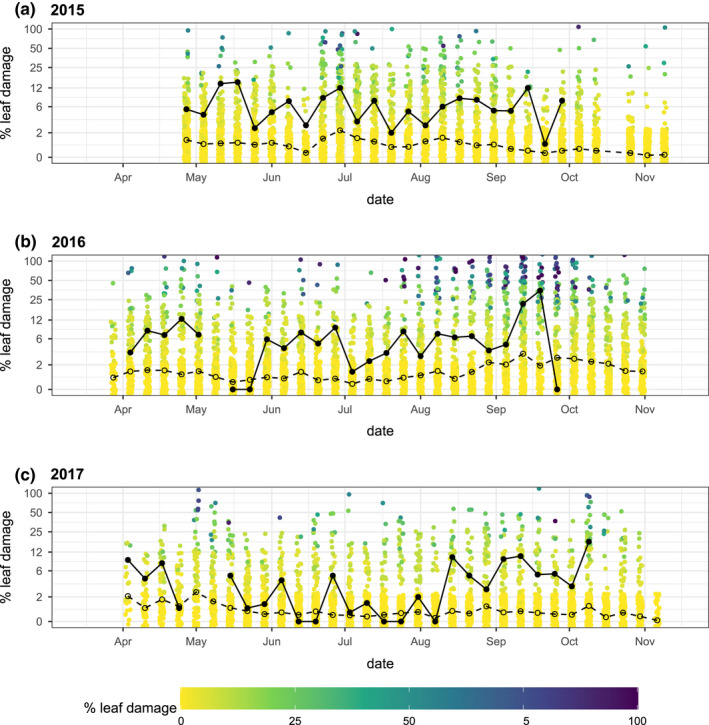
Weekly mean percent leaf damage in (a) 2015, (b) 2016, and (c) 2017. The open points and dashed line indicate the weekly mean percent leaf damage; the filled points and solid lines indicate the weekly mean percent leaf damage on plants where caterpillars were observed

**FIGURE A18:**
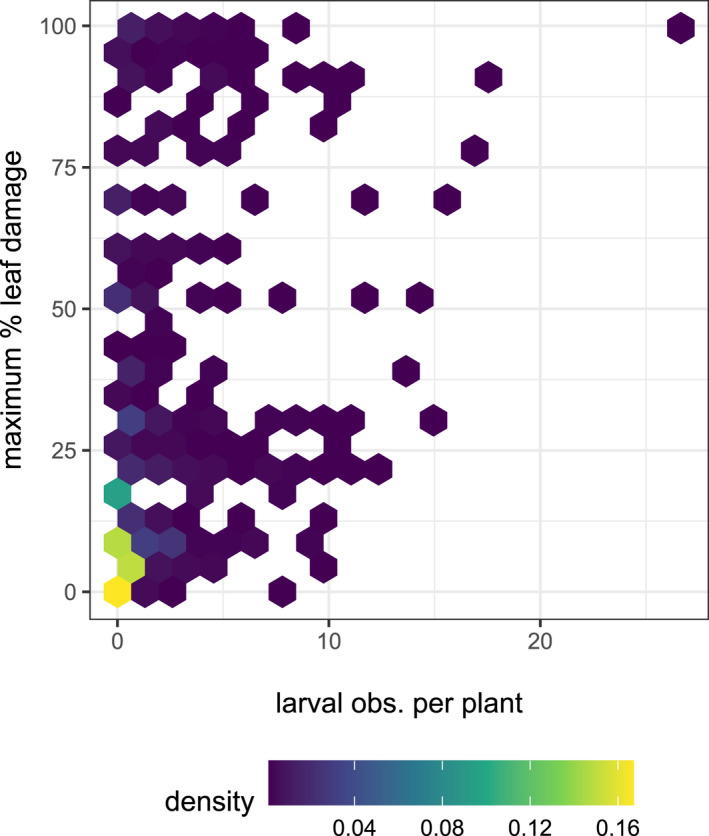
While most plants were not observed with monarchs and did not show leaf damage, milkweed plants with more larval observations per plant each year tended to have higher maximum observed leaf damage measures. Color represents the proportional density of points per hexagonal bin

The mean percent leaf area removed across the population was generally low (Figure 8), averaging 2.7% across all 3 years of this study (2015: 2.1%, 2016: 4.1%, 2017: 1.7%). Considering only observations where monarch caterpillars were present, this means increased to 6.1% (2015: 6.8%, 2016: 6.2%, 2017: 5.0%). Across all plants, the weekly mean percent leaf area removed ranged from 0.1% to 13.8%; for plants with monarch caterpillars present, this metric ranged from 0% to 34.8% (Figure 8). However, the distribution of damage estimates was strongly skewed with high variance, with some plants experiencing much higher rates of herbivory throughout each year (Figure 8). Across the study, the annual maximum leaf damage was positively correlated with the count of caterpillar observations on that plant (*r* = .34, *p* < .0001, Figure A18).

### 3.4 Community phenology

#### 3.4.1 Predatory taxa

**FIGURE A19:**
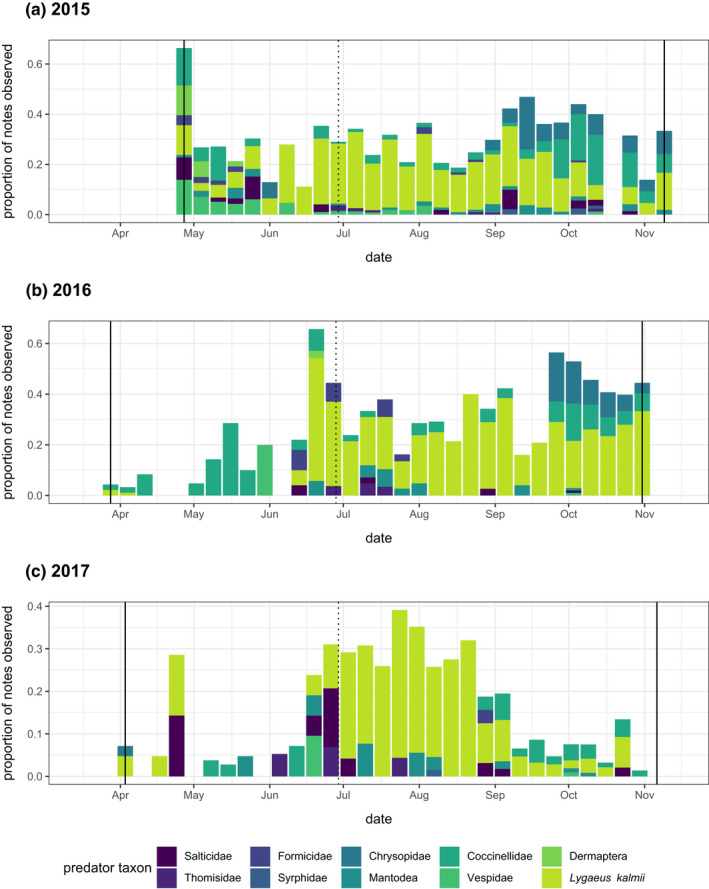
The proportion of notes that included predaceous taxa in (a) 2015, (b) 2016, and (c) 2017. Solid vertical lines indicate the start and end of observations each season. The dotted vertical line represents day 180, which is used to separate the early and late season in these analyses

Observations of predatory taxa varied across the years of this study (Figure A19). In 2015, the proportion of noted observations that included predatory species peaked at around 0.6 in the first week of the study, then showed a gradually increasing trend from approximately 0.25 to 0.45 across the season thereafter. In 2016, observations of predatory taxa increased to a peak above 0.6 in mid‐May, then showed a variable pattern ranging from approximately 0.15 to 0.4 before increasing above 0.4 from the end of September to November. In 2017, observations of predatory taxa showed a largely unimodal pattern that peaked above 0.35 in mid‐July, flanked by lower observation rates (generally <0.1) in the early and late margins of the growing season. While it did not seem to appreciably delay the growth phenology of milkweeds, the mowing disturbance on May 5, 2017, was coincident with a period of reduced predator densities, consistent with previous studies (Haan & Landis, 2019). The composition of the predator community changed differently across each year but was consistently and increasingly dominated by the omnivorous small milkweed bug (*Lygaeus kalmii*) during periods of high predator density and in the late season of each year (Figure A19).

**FIGURE 9:**
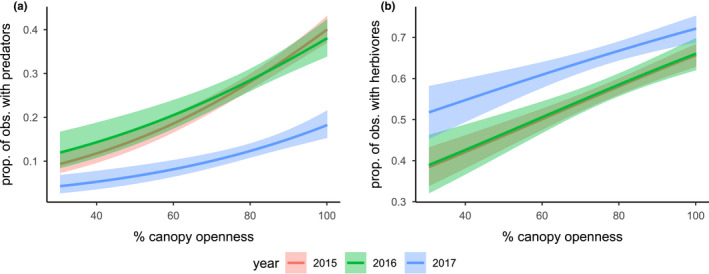
The modeled effects of canopy openness on the proportion of notes with (a) predatory taxa present and (b) herbivorous taxa present, although both predators and herbivores were more commonly observed on host plants with greater canopy openness. Among the 3 years of this study, predators were least commonly observed, and herbivores were most commonly observed in 2017. Shaded area around each fitted line indicates the 95% confidence interval

Predatory taxa were more commonly observed on milkweed host plants with greater canopy openness (*p* < .0001, Figure 9a); while these observation densities differed by year (*p* < .0001), the effect of canopy openness did not (*p* = .47). Predator observations were 52–56% less common in 2017 than in 2015 or 2016. Combining all years, our model predicts a 2.9‐fold increase in the proportion of notes that included predatory taxa across the observed range of canopy openness values (30–100%).

#### 3.4.2 Herbivorous taxa

**FIGURE A20:**
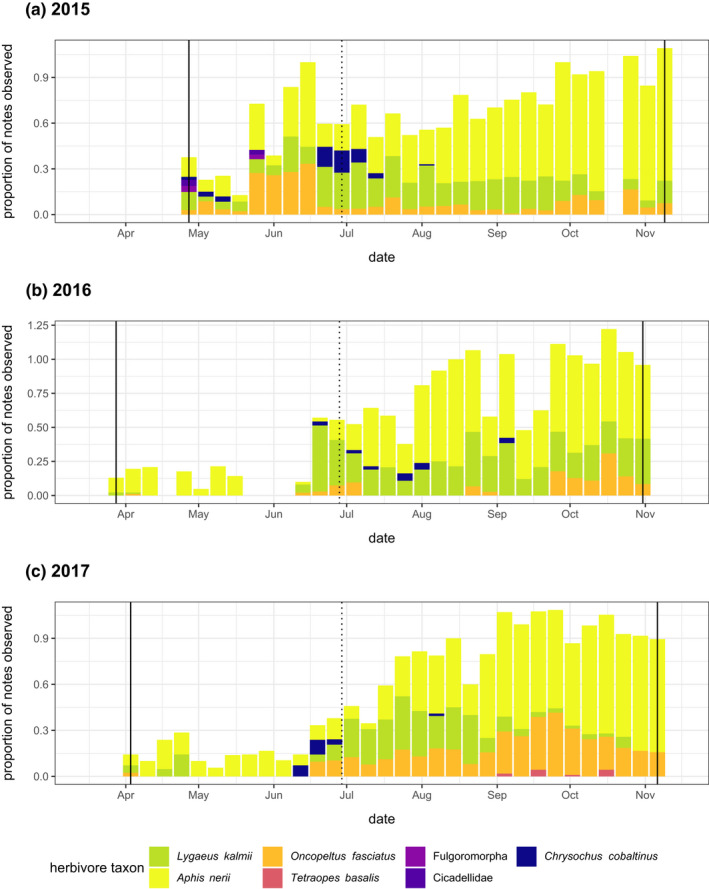
The proportion of notes that included herbivorous taxa across three seasons: (a) 2015, (b) 2016, and (c) 2017. Solid vertical lines indicate the start and end of observations each season. The dotted vertical line represents day 180, which is used to separate the early and late season in these analyses

By comparison, the community of herbivorous taxa was largely composed of oleander aphid (*Aphis nerii*), small milkweed bug (*Lygaeus kalmia*), and large milkweed bug (*Oncopeltus fasciatus*) and showed a relatively consistent pattern of increasing observation rates throughout each season (Figure A20). Although present at the beginning of each season, oleander aphid became an increasingly larger proportion of the herbivorous community composition in the late season.

Milkweed plants with greater canopy openness generally had more observations of herbivorous taxa (*p* < .0001, Figure 9b). Observations of herbivorous taxa differed by year (*p* < .0001) and were generally more common in 2017 than in 2015 or 2016. The effect of canopy openness did not differ significantly among years (*p* = .56), showing a predicted 59% increase across the observed range of canopy openness values (30–100%).

### 3.5 Combined analysis

**FIGURE 10:**
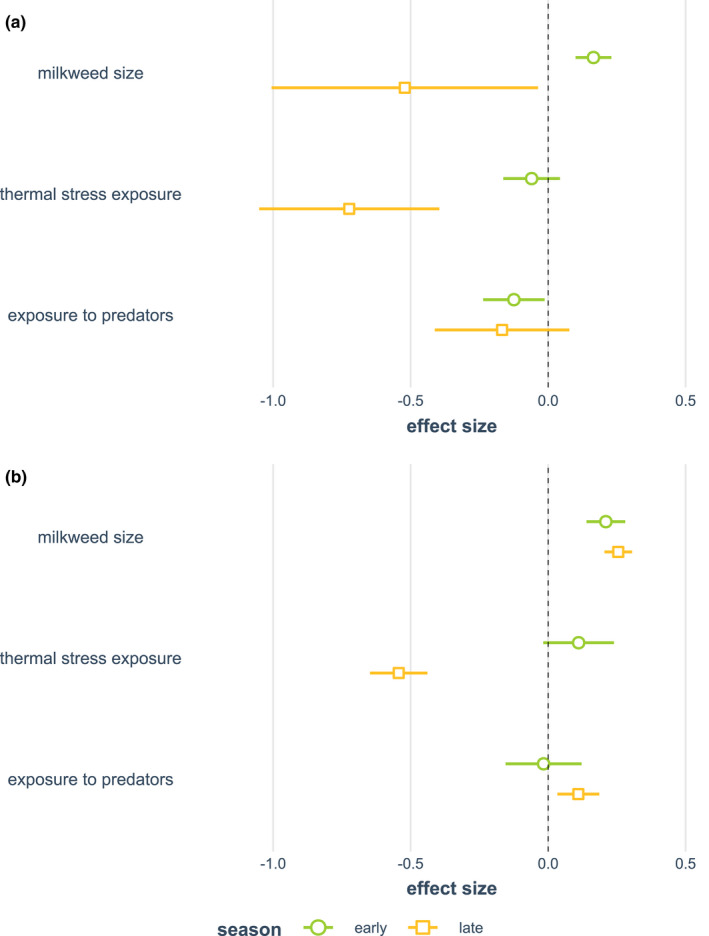
Comparison of standardized effect sizes for early‐ and late‐season GLMs of (a) egg and (b) caterpillar observation counts. Effect sizes are standardized by 1 SD, and lines represent the 95% confidence interval

A model of egg observation counts using *milkweed size* (maximum total stem length), *thermal stress exposure* (degree‐minutes ≥38°C), *exposure to predators* (proportion of notes with predators observed), *season* (early vs. late), and all pairwise interaction effects with season showed that the effect of milkweed availability differed significantly in the early and late season (*p* = .001). As a result, we analyzed early‐ and late‐season data in separate models. These models showed that milkweed availability was associated with egg counts positively in the early season and negatively in the late season (early: *p* < .0001; late: *p* = .035, Figure 10a). Thermal stress did not have a significant effect on egg counts in the early season but showed a strong negative effect in the late season (early: *p* = .25; late: *p* < .0001, Figure 10a). Additionally, exposure to predators had a negative effect on egg counts in the early season (*p* = .029, Figure 10a); this effect remained negative but not significant in the late season (*p* = .18, Figure 10a).

A parallel model of caterpillar observation counts indicated that the effects of thermal stress (*p* = .0007) varied by season. The effects of predator exposure also showed a marginally significant interaction with the season (*p* = .07). We subsequently analyzed models that considered the early and late seasons separately; these models showed significant positive effects on milkweed availability in both seasons (early: *p* < .0001; late: *p* < .0001, Figure 10b). The effects of thermal stress exposure were widely divergent in the early and late season, showing marginal positive effects in the early season (*p* < .09), followed by strong negative effects in the late season (*p* < .0001, Figure 10b). Exposure to predators had a nonsignificant negative effect in the early (*p* = .81) and a significant positive effect in the late season (*p* = .005, Figure 10b).

## 4 DISCUSSION

Our study has three key findings. First, this study documents seasonal windows of opportunity in the wild, migratory western monarch population. Second, these seasonal windows appear to be constrained by different factors in the early and late part of each breeding season. Third, climatic and microclimatic variation strongly shaped the timing and relative importance of different limiting factors in this study. Here, we examine each of these findings and consider their implications in the context of a declining western monarch population.

### 4.1 Seasonal windows of opportunity

Our results show early‐ and late‐season windows of opportunity for monarch development on narrow‐leaved milkweed (Figure 4). Although the specific timing of these windows varied from year to year, all 3 years showed 2‐ to 4‐week windows of higher recruitment in the early and late season, separated by a mid‐summer period with substantially lower developmental prospects (Figure 4). These windows do not represent the direct offspring of two successive generations, as they were separated by more than 12 weeks (Figure 4), while the total (egg to adult) development time of monarchs is generally less than 22 days (York & Oberhauser, 2002; Zalucki, 1982). Adult monarchs were present at our site throughout the breeding season and were actually commonly observed during a period of low egg and larval densities in the mid‐summer (Figure A11). Thus, the observation of seasonal windows in this study seems to suggest periods with increased recruitment potential, rather than simply reflecting a seasonal pattern of adult monarch density at our site.

However, in contrast to previous experimental studies in this system (Yang et al., 2020; Yang & Cenzer, 2020), the early‐season windows of opportunity in this study were largely unrealized; only a small proportion of these monarch eggs survived to be observed as later larval instars (Figures 4 and 5). Thus, our current study suggests an early‐season window characterized by high recruitment potential (i.e., oviposition) but ultimately low survivorship (low caterpillar observations). This difference suggests the possibility of a density‐dependent constraint on monarch success resulting from high oviposition densities in the early season. More broadly, the observed variation in monarch success between and within years suggests that the windows of opportunity for monarch development in this study were constrained by different factors in different years, and in the early and late seasons of those years.

### 4.2 Milkweed limitation, predation, and thermal stress

#### 4.2.1 Early‐season constraints

In the early season of each year, we observed a period of high oviposition density on a subset of host plants (Figures 4 and A10), with relatively low survivorship to later larval stages (Figure 5). One possible explanation for this pattern is seasonal host plant limitation. This seasonal host plant limitation could arise from a transient period where the momentary demand for host plant resources exceeds the available supply.

Phenological mismatches between the arrival of migratory monarchs and the emergence of their milkweed host plants could provide a possible mechanism for seasonal host plant limitation. Previous studies have suggested that monarchs evolved to use environmental cues that maintained phenological correspondence between their spring migration and the emergence of early‐season milkweed shoots (Guerra & Reppert, 2015; Reppert & de Roode, 2018). Cueing mechanisms to maintain this correspondence could be adaptive if optimal oviposition timing reflects a balance between the dynamic constraints of resource quality and quantity. Although these ontogenetic patterns vary by milkweed species, defensive traits such as latex exudation, trichome density, and leaf toughness generally seem to increase through the early season (Pearse et al., 2019; Yang et al., 2020). Caterpillars feeding on young plants with relatively weak defensive traits show initially higher survivorship and significantly faster growth (Yang et al., 2020). In contrast, monarch neonates feeding on mature intact plants experience high rates of mortality during initial feeding, while neonates feeding on leaves with experimentally reduced latex exudation experienced significantly higher survival and growth (Zalucki, Brower, et al., 2001; Zalucki, Malcolm, et al., 2001). These studies suggest a pattern of declining resource *quality* over time, consistent with ontogenetic patterns that have been observed in other herbaceous plants (Barton & Koricheva, 2010; Boege & Marquis, 2005). Thus, early‐season milkweeds likely provide relatively high‐quality resources with relatively weak defensive traits (Yang et al., 2020), potentially favoring earlier oviposition. However, resource *quantity* constraints create a simultaneous selection pressure in the opposite direction. Previous studies on narrow‐leaved milkweed indicate that, despite their relatively high initial survivorship, caterpillars on young host plants eventually experience reduced survivorship due to the small size of individual plants (Yang et al., 2020). Thus, milkweeds may present a phenological challenge of simultaneously declining host plant quality and increasing quantity each season; oviposition too early increases the probability of starvation, while oviposition too late incurs the developmental costs of increasing plant defensive traits (Yang et al., 2020, Yang & Cenzer, 2020).

We hypothesize that these rapidly changing milkweed traits create a dynamic landscape where ovipositing females are selecting for trait combinations that balance resource quality and quantity. We observed a patchy distribution of egg and caterpillar observations (Figure A14, Movies S1 and S2) where most monarch eggs (66%) were observed in densities of two or greater (Figure A13). Although recently emerged host plants are relatively small in the early season, both egg and caterpillar counts were highest on the largest available milkweeds in the early season (Figures 6 and 10), which tended to be associated with more open canopy environments (Figures 3 and 7, A16 and A17). This spatial patchiness was unexpected given previous evidence of conspecific avoidance (Jones & Agrawal, 2019) and the preponderance (98.7%) of plant observations without any eggs present. However, ovipositing monarchs appear to be undeterred by the presence of conspecific eggs (as opposed to caterpillars; Zalucki & Kitching, 1982), consistent with the Poisson distribution of nonzero egg and caterpillar counts. A similar pattern of oviposition has been described in other species, driven by strong host plant selection (Doak et al., 2006); in both of these cases, the vast majority of host plants were not selected for oviposition. These observations of oviposition site selectivity are also consistent with previous studies indicating that monarchs favor younger but also taller and rapidly growing host plants for oviposition (Zalucki & Kitching, 1982), and studies showing the preferential oviposition and increased developmental success of monarchs on the rapid regrowth of milkweeds following physical disturbance (Fischer, 2015; Haan & Landis, 2019). However, the concentration of herbivore demand on a small subset of selected host plants could increase the potential for competition: the subset of plants where caterpillars were present showed leaf damage estimates that were more than twice as high as the site‐wide mean (Figure 8) and these damage estimates increased with increasing caterpillar counts (Figure A18). Previous studies suggest that monarchs experience negative effects from intraspecific competition among caterpillars: Monarchs avoid oviposition on plants where caterpillars are already present (Jones & Agrawal, 2019) and show reduced survival and growth when multiple caterpillars are on the same plant (Flockhart et al., 2012; Nail, Stenoien, et al., 2015). Thus, while the preference for larger early‐season host plants observed in our study might reflect past selection pressures to reduce the risk of starvation, the resulting patchiness of monarch oviposition (Figures 6, A13 and A14, Movie S1) could also potentially exacerbate seasonal host plant limitation by concentrating monarch herbivory in space, contributing to a pattern of “limitation by selectivity.”

In addition to this concentration of herbivore demand in space, our data also suggest that monarch oviposition was concentrated in time (Figure 4), with especially high rates of oviposition during a short period in the early growing season when even the largest plants in the population were relatively small (Figure A10). A similar pattern of seasonally compressed oviposition activity has been observed in the eastern migratory range (Nail, Stenoien, et al., 2015), but the proximity of overwintering sites in the western range could increase the potential for relatively synchronized migratory arrivals and high egg densities. A pattern of more temporally compressed oviposition could also result if coastal overwintering populations are disaggregating before most inland milkweed host plants are available. While the departure timing of eastern monarchs from Mexican overwintering sites does not appear to have shifted in recent years (Stenoien et al., 2018), western monarchs have shown earlier first flight observations in association with warmer, wetter winter temperatures (Forister & Shapiro, 2003). While it seems plausible that the spring migration of western monarchs has advanced under ongoing climate change, it is unclear whether the growth phenologies of western milkweeds have kept pace. Their growth phenology has not advanced significantly in the east (Howard, 2018), and considerably less is known about the emergence phenology of the many milkweed species in the West. If the phenological advances of migrating western monarchs are increasingly mismatched with the growth phenology of their milkweed host plants, this could create the potential for an “ecological crunch” period with transiently increased resource competition (Wiens, 1977).

Thus, the concentration of herbivore pressure in space and time observed in our study suggests that landscape‐scale or season‐long mean leaf damage estimates may not provide a meaningful metric of milkweed limitation. The relevant spatial and temporal scales may be smaller, requiring a more detailed, spatially and temporally explicit approach that considers seasonal and developmental changes in plant traits and spatiotemporal variation in herbivore demand.

In addition to these bottom‐up factors, top‐down factors could also contribute to the pattern of reduced early‐season survival. Previous studies have consistently documented the strong effects of diverse natural enemies on the survivorship of monarch eggs and caterpillars, especially in early‐life stages (De Anda & Oberhauser, 2015; Hermann et al., 2019; Oberhauser, 2012; Oberhauser et al., 2015; Prysby, 2004). While our study documented seasonal patterns of predator observations (Figure A19), these patterns varied widely between years and did not show a consistent pattern of greater predator densities in the early season. However, we speculate that the small size of host plants and the sparseness of surrounding vegetation in the early season could expose monarchs to greater predation risk (cf., Strauss & Cacho, 2013). Future studies will be necessary to separate the effects of resource competition and predation in the early season.

#### 4.2.2 Late‐season constraints

We observed significantly lower densities of eggs and significantly higher densities of caterpillars in the late season (Figures 4 and 5), with substantial inter‐ and intra‐annual variation in the timing and magnitude of the late‐season windows. We hypothesize that these patterns may have been associated with the combined effects of direct thermal stress (Figures 1 and A4) and changing host plant defensive traits (Yang et al., 2020) but were unlikely to be constrained by the total availability of milkweed biomass (Figures 2 and 8).

Exposures to stressful temperatures were much higher in the late season than in the early season (Figures 1 and A4), and our model combining bottom‐up (milkweed size), abiotic (thermal stress), and top‐down (predator) explanatory factors showed a strong negative effect of thermal stress in the late season for both eggs and caterpillars (Figure 10). We speculate that the increasing incidence and intensity of heatwaves may have reduced the developmental success of monarchs. This interpretation is consistent with observed interannual variation in the effect of canopy openness: Milkweeds in more open canopy environments generally emerged earlier (Figure 3a), grew faster (Figure 3b), attained larger maximum sizes (Figure 4c), and attracted higher monarch egg densities (Figure 7a); however, these same host plants uniquely did not support higher densities of caterpillars in 2017 (Figure 7b). Whereas 2016 saw high egg and caterpillar densities across the site, caterpillar observations in 2017 were largely restricted to the cooler, shadier sections of our field site with reduced canopy openness (Figures A14 and A15, Movies S1 and S2). In comparison, cooler late‐season conditions in 2016 seemed to allow greater late‐season larval success in more open environments (Figure A14).

The 2017 growing season followed a wet winter and the termination of a multi‐year drought (Figure A2); the milkweeds in this year were phenologically delayed but grew to be significantly larger in comparison to the other 2 years of this study (Figure 2). Both milkweed senescence and the late‐season window of opportunity were delayed in 2017 (Figures 2c and 4c), suggesting that these late‐season windows of opportunity may be structured around seasonal reductions in plant defensive traits. The larger size of milkweeds in 2017 seems unlikely to have limited monarch developmental success by itself; in all years of this study, larger milkweed plants were generally associated with *higher* caterpillar observation densities (Figure 7f). This generally positive relationship between plant size and larval success is consistent with reduced competition on larger plants (Flockhart et al., 2012; Nail, Stenoien, et al., 2015). Although our analyses lacked sufficient egg observations to detect significant selectivity in the late season, the observed trend suggests that female monarchs may have shifted from favoring relatively larger plants in the early season towards favoring relatively smaller plants in the late season (Figure 6), consistent with a greater emphasis on resource quality over quantity. This interpretation is also consistent with the negative effect of milkweed size on late‐season egg counts observed in our overall analysis (Figure 10a).

The significantly higher ratio of fifth instar larval observations to eggs in the late season (Figure 5) suggests relatively higher rates of survival in the late season. These observation ratios provide a relative metric of survivorship for comparisons across years and seasons but should not be interpreted as absolute measures of daily or stage‐specific survivorship. Absolute measures of survivorship generally require individually identified eggs and larvae (e.g., De Anda & Oberhauser, 2015; Nail, Batalden, et al., 2015; Yang et al., 2020); for observational survey studies, estimating survivorship requires additional assumptions due to inherent differences in the duration and detectability of life stages (Grant et al., 2020). However, our ability to compare seasonally relative metrics of survivorship (i.e., observation ratios) does not rely on complete detection or other model assumptions. Specifically, because the goal of this analysis was to compare relative changes in monarch survivorship within and across three breeding seasons, differences in the detectability of life stages do not present a problem unless there is also a strong seasonal pattern in this detectability bias. While we were initially concerned that we might have a lower ability to detect eggs on larger plants in the late season, we actually observed more eggs on larger plants overall (Figure 7e). Moreover, the difference between the early‐ and late‐season observation ratios persists in analyses that use more detectable early‐instar caterpillars instead of eggs (Figure A12), and in years with substantially smaller plant sizes where one might expect greater overall detection (Figure 2). Thus, while no study can completely eliminate the possibility of missed observations, seasonal detection bias alone seems unlikely to explain early‐ and late‐season differences in monarch observation ratios. Consistent with this, previous studies have used similar observation ratios as relative metrics of survivorship without adjusting for seasonal or stage‐specific differences in detectability (Nail, Stenoien, et al., 2015; Oberhauser et al., 2001).

While natural enemies (predators, parasitoids, parasites, pathogens) are known to strongly limit the survivorship of monarch eggs and caterpillars generally (Altizer & Oberhauser, 1999; Hermann et al., 2019; Oberhauser, 2012; Oberhauser et al., 2015; Prysby, 2004), their specific role in constraining late‐season windows of opportunity is less clear. Observations of predaceous taxa were highly variable between and within years, both in terms of their proportion of noted observations and their taxonomic composition (Figure A19). The relative importance of seasonal variation in predation pressure relative to other constraints on caterpillar recruitment (e.g., initial oviposition density and resource limitations) remains uncertain, and future experimental studies will be necessary to examine the relative contribution of abiotic (climatic), bottom‐up (host plant‐mediated) and top‐down (natural enemy) constraints.

Interactions with other herbivores could also have affected late‐season windows of opportunity in our study. In all 3 years of this study, observations of other herbivore taxa increased from relatively low densities (<0.3) in the early season to relatively high densities (>0.9) by the end of the late season (Figure A20). This seasonal pattern was mostly driven by increased observations of oleander aphid (*Aphis nerii*). Oleander aphids have been previously shown to positively affect the growth of monarch caterpillars via induced changes in the defensive traits of milkweed host plants (Ali & Agrawal, 2014), though these same changes also increase the virulence and transmission of *Ophryocystis elektroscirrha*, a monarch‐specific protozoan parasite (de Roode et al., 2011). While the net effects of these countervailing interactions during our study are unknown, our data suggest that important interactions with other herbivores in the community are likely to be more common in the late season than in the early season of each year. In particular, the near‐ubiquitous presence of oleander aphids in the late season could potentially hasten host plant senescence, advancing the late‐season window of opportunity for monarchs.

### 4.3 The fundamental effects of climate

The climatic effects observed in our study were both complex and fundamental, suggesting specific effect pathways that varied across the early and late seasons of each year. In addition to their direct abiotic effects, this climatic (and microclimatic) variation likely played a fundamental role in setting the stage for subsequent biotic interactions. For example, while drought conditions limited milkweed growth they also advanced milkweed phenology (Figures 2 and A9), and may have increased foliar nitrogen and reduced key defensive traits (Couture et al., 2015). In our study, drought conditions did not seem to limit monarch success in any simple sense and may have had their strongest effects via changes in host plant phenology and quality, rather than productivity. Similarly, warmer later winter temperatures were associated with advanced milkweed phenology in the early season (Figures 2 and A3) but may have also increased latex exudation (Couture et al., 2015) and exposure to stressful temperatures in the late season (Figures 1 and 10, and A4). While experiments will be necessary to assess causation, our study suggests the value of a high‐resolution seasonal perspective to understand changing climatic effects between and within years.

Our analysis of canopy openness further illustrates the fundamental but complex role of climatic variation on the individual plant scale. Plants in open canopy environments showed earlier growth phenologies and attained larger sizes (Figure 3); in turn, these earlier, larger host plants were preferentially selected for early‐season oviposition (Figure 6) and supported more larval observations per plant overall (Figure 7). These biotic responses likely reflect abiotic drivers—the greater direct light exposure, daytime heating, and soil drying associated with more open canopy environments. Conversely, monarchs in more exposed locations may also have experienced greater direct thermal stress during years with more intense heatwaves (Figure 7b), and higher predator and herbivore densities (Figure 9). These patterns illustrate the complex pathways by which microclimatic variation can affect monarch development but also suggest that microhabitat variability in heterogeneous habitats could buffer species interactions under changing climatic conditions (e.g., Rytteri et al., 2021).

Disentangling the direct and indirect (i.e., mediated via the host plant or the surrounding community) effects of climatic variation on monarch development is likely to be difficult (Boege & Marquis, 2006; Despland, 2018; Kharouba & Yang, 2021) but a seasonal perspective could help. Our study illustrates how multiple factors interact to constrain the developmental success of monarchs, with important differences in their relative contributions in the early and late seasons. A key message of this work is that it may be more relevant to recognize the fundamental role of climatic drivers in shaping subsequent interactions in this system rather than attempting to compare the relative effects of climatic and other factors as strictly alternative explanations. In this view, abiotic climate drivers are not strict alternatives to biotic hypotheses; the climate is a fundamental driver with both direct effects and broad indirect effects mediated by the biotic community, including changes in the timing, quality, and quantity of milkweed host plants. This study highlights the importance of developing temporally explicit, sequential hypotheses (Yang, 2020; Yang et al., 2021) to examine how climatic variation shapes the seasonal timing and magnitude of abiotic, bottom‐up, and top‐down constraints on species interactions.

### 4.4 Context and conclusions

The 3 years of this study (2015–2017) document the last 3 years of the western monarch population prior to the precipitous 86% single‐year population decline in 2018 (Pelton et al., 2019). Throughout this study, the overwintering western monarch population was estimated to be on the order of 200–300 thousand butterflies (Crone et al., 2019; Schultz et al., 2017). In the winter of 2018–2019, the overwintering western monarch population was below the estimated extinction threshold of 30,000 overwintering butterflies (Pelton et al., 2019; Schultz et al., 2017), a rapid decline that continued to an overwintering population of approximately 2000 butterflies in the winter of 2020–2021 (Crone & Schultz, 2021; James, 2021) before an unexpected approximately 100‐fold population increase in 2021. These broader population trends offer important context for the patterns observed in our current study, and keenly illustrate the limits of our current understanding. The variability of recent years belies a broader multi‐decadal declining trend in the western monarch population (Espeset et al., 2016; Schultz et al., 2017) but also suggests the potential for both unexpectedly rapid population declines and increases in this system. The findings of our study show how sequences of favorable or unfavorable seasonal conditions can strongly affect monarch recruitment and may be relevant to understand recent population variability. While unfavorable conditions in either the early or late season can constrain recruitment, years that combine uncommonly favorable conditions across the breeding season have the potential for rapid, compounding population increases.

The results of this study suggest that climatic variation among years and across seasons plays a foundational role in the timing and success of monarch developmental windows. These results seem to be in contrast to previous continental‐scale modeling efforts that did not detect a strong signal of climatic factors in historic monarch population declines in the east (Flockhart et al., 2015; Stenoien et al., 2018; Zalucki et al., 2015), though climate factors have been associated with phenology and growth of the monarch population in specific parts of the eastern range (Zipkin et al., 2012; Zylstra et al., 2021). In comparison, studies in the western range generally suggest a stronger role for climatic factors, though the relative contributions of climatic and nonclimatic factors have been difficult to separate (Crone et al., 2019; Espeset et al., 2016; Stevens & Frey, 2010). Our current study differs from these previous studies in aim, approach, scale, and timing. A key difference is that our study does not aim to explain historical, continental‐scale patterns of monarch abundance, nor to evaluate all plausible constraints on monarch developmental success. Our emphasis on within‐season dynamics at a local scale allowed us to examine climatic and microclimatic drivers of milkweed phenology and growth across years, across seasons within years, and at the scale of individual plants. If the effects of climatic drivers are strongly region‐ or season‐specific, the effects observed in this study might be difficult to detect in studies that integrate across larger spatial or temporal scales. Our study also benefited from past experimental studies into the thermal biology of monarchs (Nail, Batalden, et al., 2015; York & Oberhauser, 2002; Zalucki, 1982), which allowed us to infer lethal and sublethal thermal constraints. In addition, it is also possible that the timing of our study allowed us to observe the effects of direct thermal stress that have become more apparent in recent years. Nine of the ten warmest years in the global record occurred in the past decade (National Centers for Environmental Information, 2021), and the frequency and intensity of heatwave events have continued to increase globally (IPCC, 2021) and in California (Gershunov & Guirguis, 2012).

Our findings are consistent with previous studies suggesting seasonally specific limits on monarch recruitment (Espeset et al., 2016; Zipkin et al., 2012), with particular emphasis on the early season (Crone et al., 2019; Espeset et al., 2016; Zylstra et al., 2021). However, while Espeset et al. (2016) and Crone et al. (2019) interpreted early‐season declines as stemming from reduced immigration from overwintering aggregations, our study was focused on the development of eggs and caterpillars in the breeding range. Throughout this study, we observed strong breeding activity but consistently low survival of eggs and early‐instar caterpillars in the early season (Figure 4). In contrast, Espeset et al. (2016) analyzed observations from 1972 to 2014 that did not find evidence for monarch breeding activity at low‐elevation sites prior to May and did not detect phenological shifts in the arrival of monarchs. Our observations of significant breeding activity prior to May suggest that some advancement in local breeding activity may have occurred in recent years. Consistent with this, previous studies have detected advances in the first flight observations of monarchs associated with warmer and wetter spring conditions (Forister & Shapiro, 2003). Moreover, our observations of reduced breeding success in the early season and more successful caterpillar production in the late summer and early fall are intriguingly different from the pattern of spring and summer breeding that has historically been observed in the Central Valley (Art Shapiro, *pers. comm*.). While the causes and consequences of these changing seasonal dynamics remain unclear, we speculate that changes in the success of early‐season breeding could have especially large demographic consequences in the context of an expanding, multi‐generation summer migratory population.

Previous studies have suggested that warmer winter and spring conditions generally favor monarchs (Espeset et al., 2016, Zipkin et al., 2012). Our analysis has a limited ability to assess this pattern, but our observations are at least partly consistent; substantially higher monarch observations occurred during a year with marginally warmer early‐spring conditions (Figures 5 and A3), and individual plants with warmer, more open canopies were generally associated with more monarch observations (Figure 7). We suggest that these patterns may be due to climate‐driven advances in the growth phenology of host plants; climatic conditions that allow for earlier host plant growth were associated with improved monarch success, potentially by increasing the temporal overlap between consumer demand and resource availability. Future experimental studies will be necessary to evaluate this hypothesis. However, our study also suggests an important caveat about the emergence of potentially stressful high temperatures in the late season; while warmer winter and spring conditions could improve the developmental prospects of monarchs by advancing the phenology of milkweed growth, subsequent summer heatwaves could limit the success of monarchs the late season. This interpretation is consistent with Zipkin et al. (2012), which noted that the otherwise positive associations between temperature and monarch development in the eastern population were not present at the warmest sites, and with Forister et al. (2021), which detected reduced observations of multiple western butterfly species in hot and dry years. This caveat is also echoed in our analysis of microclimatic variation, where greater canopy openness was associated with more caterpillar observations in 2015 and 2016 but not in 2017 (Figure 7b), a year characterized by a greater frequency and intensity of heatwaves (Figures 1 and A4).

Our observations suggest that winter and spring precipitation likely affects the timing, quality, and quantity of host plants in the early season, with delayed effects on the late‐season window of opportunity. Our observations are at odds with the positive association between early‐spring precipitation and monarch observations in other studies (Espeset et al., 2016, Zipkin et al., 2012); we observed the highest number of monarch eggs and caterpillars under persistent drought conditions, and substantially reduced monarch observation densities in a subsequent wet year (Figures 5 and A2). However, our findings are consistent with the observation of generally advancing phenologies and increasing abundances of diverse butterfly communities in this region in response to previous drought conditions (Forister et al., 2018). Our study could help to resolve these apparently conflicting findings, as these differences are consistent with the complex, combined effects of temperature and precipitation on milkweeds and monarchs, and spatiotemporal differences in their effects. In our study, cooler and wetter early‐spring conditions (as in 2017) were associated with delayed milkweed growth but ultimately larger plants and delayed senescence (Figure 2). When combined with the effects of late‐season heatwaves, these phenological delays and the increased defensive traits of larger plants (Couture et al., 2015; Yang et al., 2020) may have contributed to reduced late‐season success of monarchs even under wetter, more productive conditions. In contrast, the combination of dry spring conditions and the reduced intensity and frequency of summer heatwaves in 2016 was associated with advanced host plant phenology in the early season and increased survival in the late season. Thus, we speculate that the strongest impacts of precipitation on monarch recruitment may occur via changes in the timing of host plant growth and senescence, with specific effects that likely depend on the resulting overlap of resource availability and consumer demand, and the timing of subsequent abiotic constraints.

The degree to which monarchs experience seasonal host plant limitation more broadly remains unclear. In nature, the possibility of seasonal host plant limitation depends on the phenology of monarch migration (Dingle et al., 2005; Forister & Shapiro, 2003) relative to the phenology of milkweed emergence (Howard, 2018; Pearse et al., 2019; Yang & Cenzer, 2020), and the interacting effects of milkweed densities (Flockhart et al., 2015; Stenoien et al., 2015; Zalucki & Lammers, 2010), monarch densities (Flockhart et al., 2012; Nail, Stenoien, et al., 2015; Stenoien et al., 2015), and host plant selection behavior (Jones & Agrawal, 2019; Zalucki & Kitching, 1982). Moreover, the phenology, diversity, and distribution of milkweed host plants are substantially different in the western range of monarchs compared with the east. The high density and low success of eggs observed on relatively small plants in the early season (Figures 4 and 5, A10) are consistent with limitations in the size and availability of favored host plants during a window of high oviposition density in the early season but could also be driven by the interactive or independent effects of early‐season predation, disease or other top‐down factors. For example, the observed spatiotemporal clustering of immature monarchs on a relatively small subset of host plants could also increase their potential for parasite transmission (Lindsey et al., 2009) beyond expectations based on overall landscape‐scale estimates of milkweed availability (e.g., Spaeth et al., 2022). However, selective monarch oviposition (Figure 6) could suggest a mechanism for host limitation consistent with the appearance of generally high milkweed availability and low herbivory (Figure 8). Future studies will be necessary to evaluate the degree to which seasonal host plant limitation is occurring in the western range and the specific mechanisms that might contribute to this limitation.

The unique value of this current study emerges from the explicit examination of seasonality, which required repeated observations with high temporal resolution. This high‐resolution observational approach provided a way to examine seasonal and density‐dependent dynamics while also developing temporally explicit, sequential hypotheses to guide future studies. We hope that these efforts improve our understanding of the factors that constrain monarch development across the season, and the potential for future population resilience.

## AUTHOR CONTRIBUTIONS

**Louie Yang:** Conceptualization (lead); data curation (lead); formal analysis (lead); funding acquisition (lead); investigation (lead); methodology (lead); project administration (lead); resources (lead); software (lead); supervision (supporting); validation (lead); visualization (lead); writing – original draft (lead); writing – review and editing (lead). **Karen Swan:** Investigation (supporting); project administration (supporting); resources (supporting); supervision (supporting). **Eric Bastin:** Project administration (supporting); resources (supporting); supervision (supporting). **Jessica Aguilar:** Investigation (supporting); project administration (supporting); supervision (supporting). **Meredith Cenzer:** Supervision (supporting); writing – review and editing (supporting). **Andrew Codd:** Investigation (supporting); project administration (supporting); resources (supporting); supervision (supporting). **Natalie Gonzalez:** Investigation (supporting). **Tracie Hayes:** Software (supporting). **August Higgins:** Project administration (supporting). **Xang Lor:** Investigation (supporting); project administration (supporting); supervision (supporting). **Chido Macharaga:** Supervision (supporting). **Marshall Sumner McMunn:** Software (supporting); writing – review and editing (supporting). **Kenya Oto:** Data curation (supporting); investigation (supporting); project administration (supporting); supervision (supporting). **Nicholas Winarto:** Investigation (supporting); supervision (supporting). **Darren Wong:** Investigation (supporting); supervision (supporting). **Tabatha Yang:** Project administration (supporting). **Numan Afridi:** Investigation (supporting). **Sarah Aguilar:** Investigation (supporting). **Amelia Allison:** Investigation (supporting). **Arden Ambrose‐Winters:** Investigation (supporting). **Edwin Amescua:** Investigation (supporting). **Mattias Apse:** Investigation (supporting). **Nancy Avoce:** Investigation (supporting). **Kirstin Bastin:** Investigation (supporting). **Emily Bolander:** Investigation (supporting). **Jessica Burroughs:** Investigation (supporting). **Cristian Cabrera:** Investigation (supporting). **Madeline Candy:** Investigation (supporting). **Ariana Cavett:** Investigation (supporting). **Melina Cavett:** Investigation (supporting). **Lemuel Chang:** Investigation (supporting). **Miles Claret:** Investigation (supporting). **Delaney Coleman:** Investigation (supporting). **Jacob Concha:** Investigation (supporting). **Paxson Danzer:** Investigation (supporting). **Joe DaRosa:** Investigation (supporting). **Audrey Dufresne:** Investigation (supporting). **Claire Duisenberg:** Investigation (supporting). **Allyson Earl:** Investigation (supporting). **Emily Eckey:** Investigation (supporting). **Maddie English:** Investigation (supporting). **Alexander Espejo:** Investigation (supporting). **Erika Faith:** Investigation (supporting). **Amy Fang:** Investigation (supporting). **Alejandro Gamez:** Investigation (supporting). **Jackelin Garcini:** Investigation (supporting). **Julie Garcini:** Investigation (supporting). **Giancarlo Gilbert‐Igelsrud:** Investigation (supporting). **Kelly Goedde‐Matthews:** Investigation (supporting). **Sarah Grahn:** Investigation (supporting). **Paloma Guerra:** Investigation (supporting). **Vanessa Guerra:** Investigation (supporting). **Madison Hagedorn:** Investigation (supporting). **Katie Hall:** Investigation (supporting). **Griffin Hall:** Investigation (supporting). **Jake Hammond:** Investigation (supporting). **Cody Hargadon:** Investigation (supporting). **Victoria Henley:** Investigation (supporting). **Sarah Hinesley:** Investigation (supporting). **Celeste Jacobs:** Investigation (supporting). **Camille Johnson:** Investigation (supporting). **Tattiana Johnson:** Investigation (supporting). **Zachary Johnson:** Investigation (supporting). **Emma Juchau:** Investigation (supporting). **Celeste Kaplan:** Investigation (supporting). **Andrew Katznelson:** Supervision (supporting). **Ronja Keeley:** Investigation (supporting). **Tatum Kubik:** Investigation (supporting). **Theodore Lam:** Investigation (supporting). **Chalinee Lansing:** Investigation (supporting). **Andrea Lara:** Investigation (supporting). **Vivian Le:** Supervision (supporting). **Breana Lee:** Investigation (supporting). **Kyra Lee:** Investigation (supporting). **Maddy Lemmo:** Investigation (supporting). **Scott Lucio:** Investigation (supporting). **Angela Luo:** Investigation (supporting). **Salman Malakzay:** Investigation (supporting). **Luke Mangney:** Investigation (supporting). **Joseph Martin:** Investigation (supporting). **Wade Matern:** Investigation (supporting). **Byron McConnell:** Investigation (supporting). **Maya McHale:** Investigation (supporting). **Giulia McIsaac:** Investigation (supporting). **Carolanne McLennan:** Investigation (supporting). **Stephanie Milbrodt:** Investigation (supporting). **Mohammed Mohammed:** Investigation (supporting). **Morgan Mooney‐McCarthy:** Investigation (supporting). **Laura Morgan:** Supervision (supporting). **Clare Mullin:** Investigation (supporting). **Sarah Needles:** Investigation (supporting). **Kayla Nunes:** Investigation (supporting). **Fiona O'Keeffe:** Investigation (supporting). **Olivia O'Keeffe:** Investigation (supporting). **Geoffrey Osgood:** Investigation (supporting); supervision (supporting). **Jessica Padilla:** Investigation (supporting). **Sabina Padilla:** Investigation (supporting). **Isabella Palacio:** Investigation (supporting). **Verio Panelli:** Investigation (supporting). **Kendal Paulson:** Investigation (supporting). **Jace Pearson:** Investigation (supporting). **Tate Perez:** Investigation (supporting). **Brenda Phrakonekham:** Investigation (supporting). **Iason Pitsillides:** Investigation (supporting). **Alex Preisler:** Investigation (supporting). **Nicholas Preisler:** Investigation (supporting). **Hailey Ramirez:** Investigation (supporting). **Sylvan Ransom:** Investigation (supporting). **Camille Renaud:** Investigation (supporting). **Tracy Rocha:** Investigation (supporting). **Haley Saris:** Investigation (supporting). **Ryan Schemrich:** Data curation (supporting); investigation (supporting). **Lyla Schoenig:** Investigation (supporting). **Sophia Sears:** Investigation (supporting). **Anand Sharma:** Investigation (supporting). **Jessica Siu:** Investigation (supporting). **Maddie Spangler:** Investigation (supporting). **Shaili Standefer:** Investigation (supporting). **Kelly Strickland:** Investigation (supporting). **Makaila Stritzel:** Investigation (supporting). **Emily Talbert:** Investigation (supporting). **Sage Taylor:** Investigation (supporting). **Emma Thomsen:** Investigation (supporting). **Katrina Toups:** Investigation (supporting). **Kyle Tran:** Investigation (supporting). **Hong Tran:** Supervision (supporting). **Maraia Tuqiri:** Investigation (supporting). **Sara Valdes:** Investigation (supporting). **George VanVorhis:** Investigation (supporting). **Sandy Vue:** Investigation (supporting). **Shauna Wallace:** Investigation (supporting). **Johnna Whipple:** Investigation (supporting). **Paja Yang:** Investigation (supporting). **Meg Ye:** Investigation (supporting). **David Yo:** Investigation (supporting). **Yichao Zeng:** Investigation (supporting).

## CONFLICT OF INTEREST

The authors' contributions are described according to ANSI/NISO standard Contributor Roles Taxonomy (CRediT) in the Supporting Information. The authors declare that they have no conflicts of interest.

### OPEN RESEARCH BADGES

This article has earned an Open Data badge for making publicly available the digitally‐shareable data necessary to reproduce the reported results. The data is available at https://datadryad.org/stash/share/fVO1aEVjWFOmKlT9QN7dDVbS2hANUTB1IlKfrY7XHEE.

## Supporting information


Movie S1

Movie S2

CRediT authorship

## Data Availability

These data have been submitted to Dryad, and are available at this DOI: http://10.0.98.250/B8ZH1Q.
